# Supplementary material for: Overlapping Yet Distinct Functions of Vacuole Membrane Protein 1 and Transmembrane Protein 41B in Modulating Hepatic Lipoprotein Secretion and Autophagy
Source: Cell Mol Gastroenterol Hepatol. 2026 Feb 2;20(6):101751. doi: 10.1016/j.jcmgh.2026.101751 (PMC13090722; doi:10.1016/j.jcmgh.2026.101751)

Figure 1F

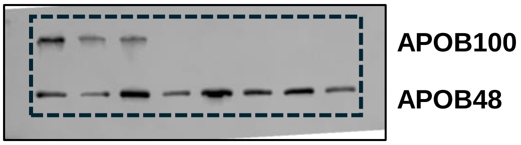

Figure 2E

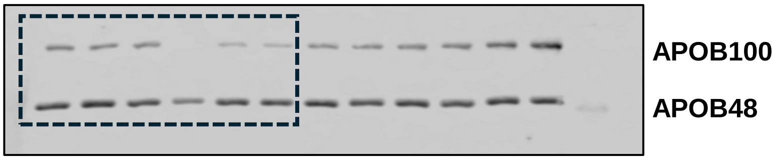

Figure 3F

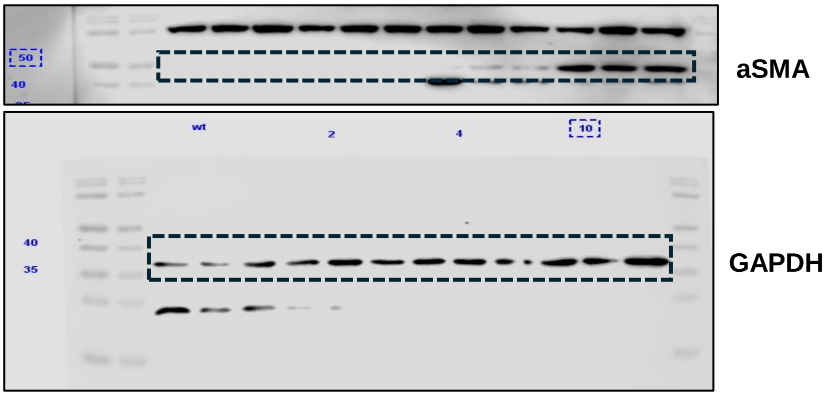

Figure 4A

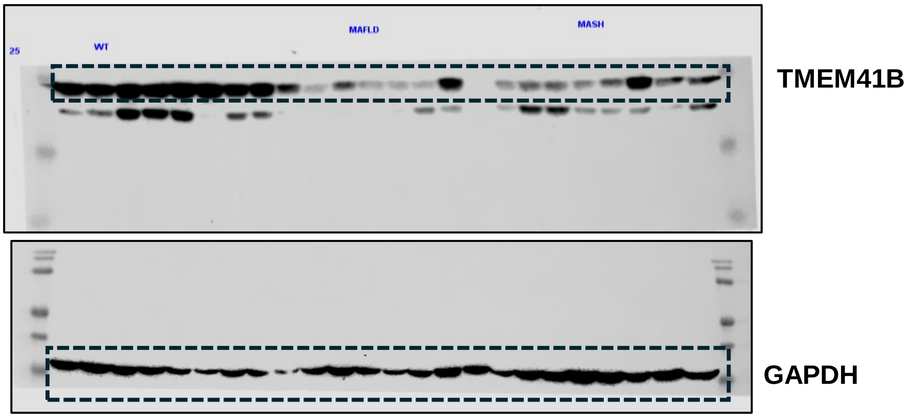

Figure 4C

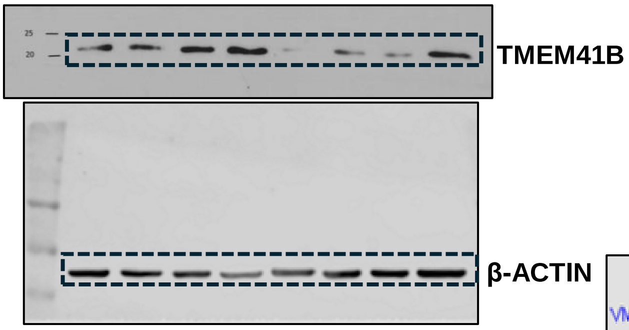

Figure 4G

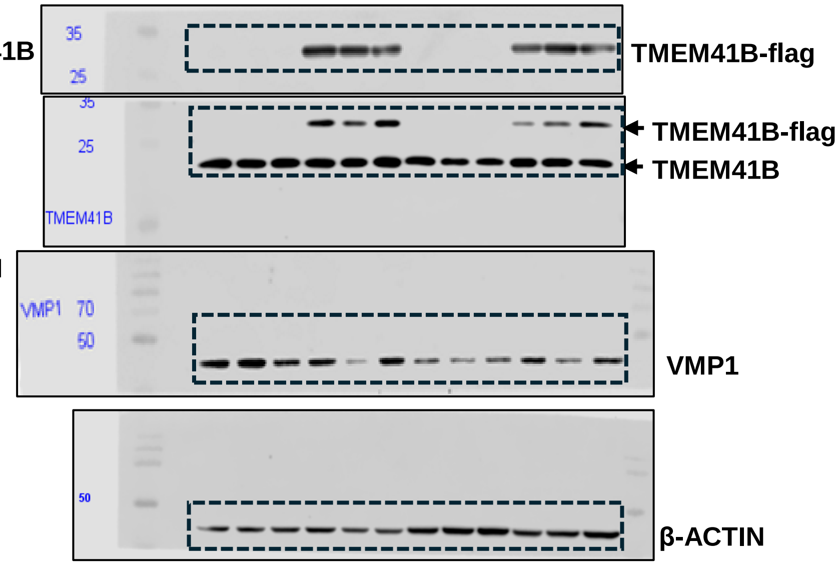

Figure 7D

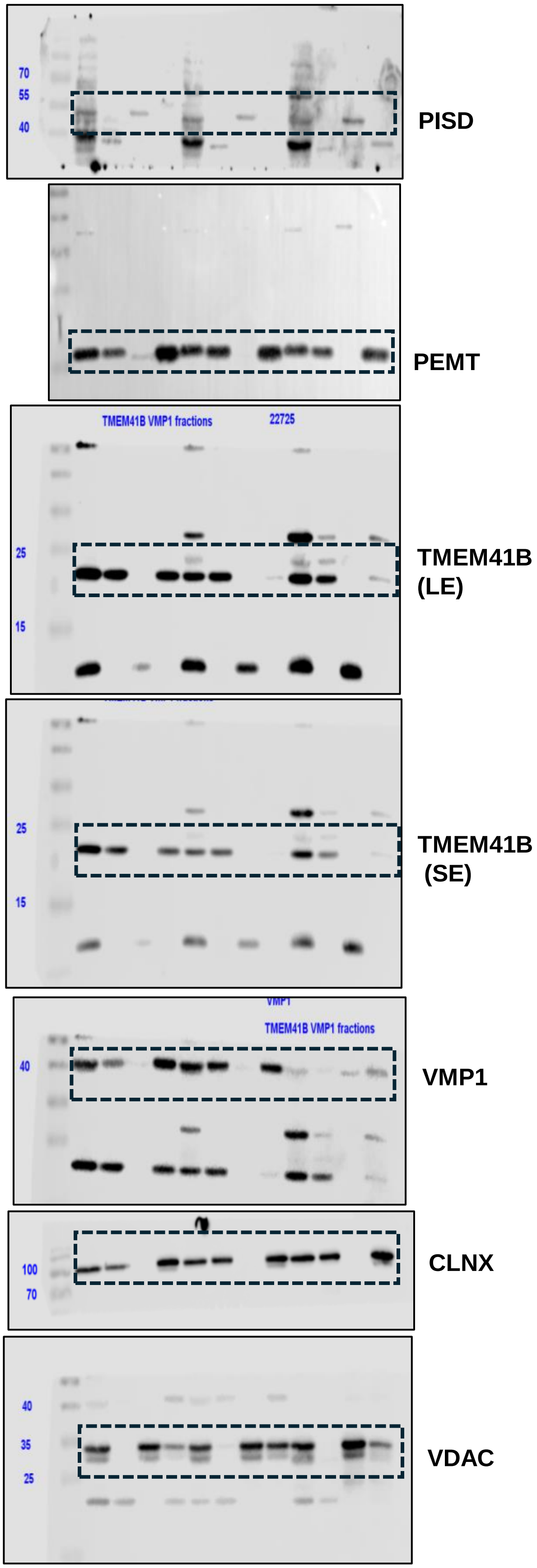

Figure 8E

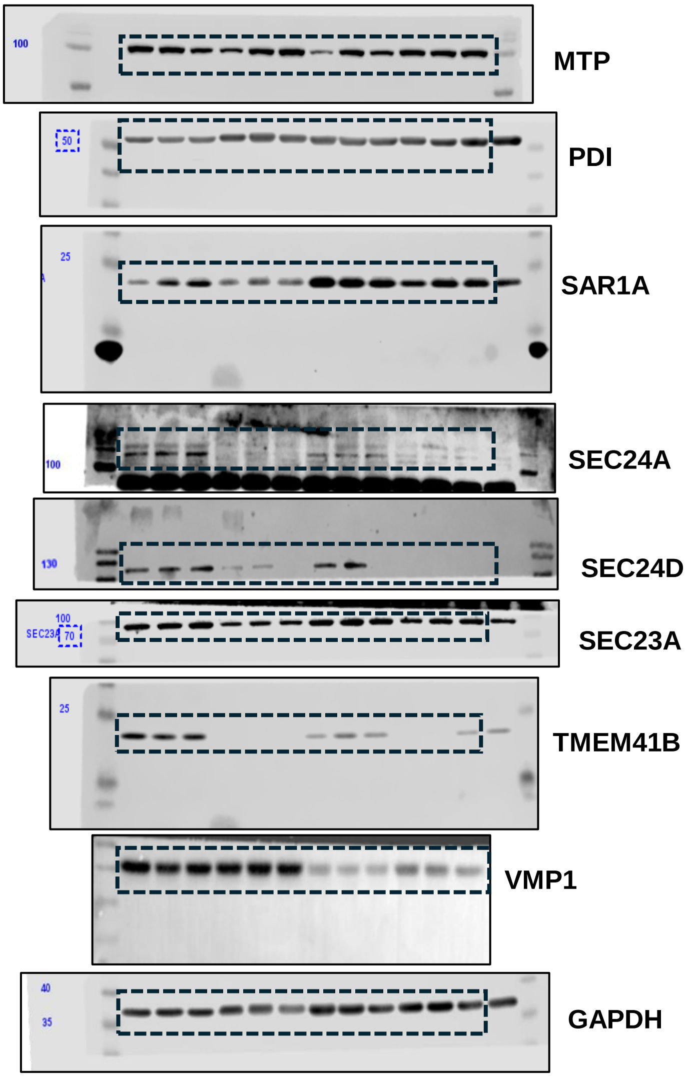

Figure 8F

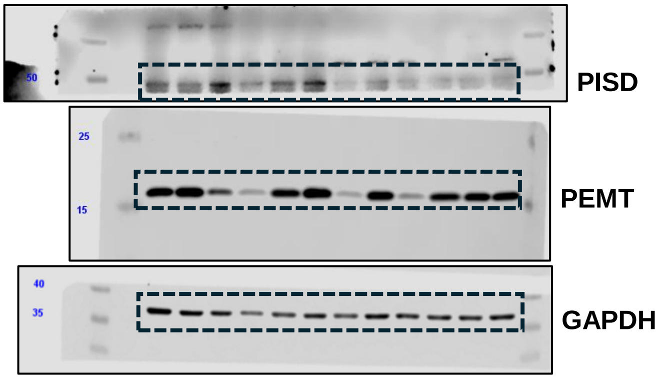

Figure 12A

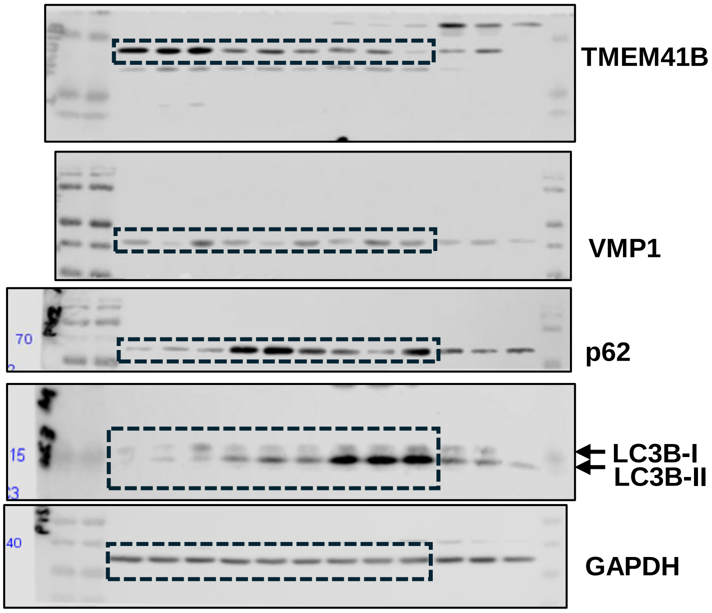

Figure 12B

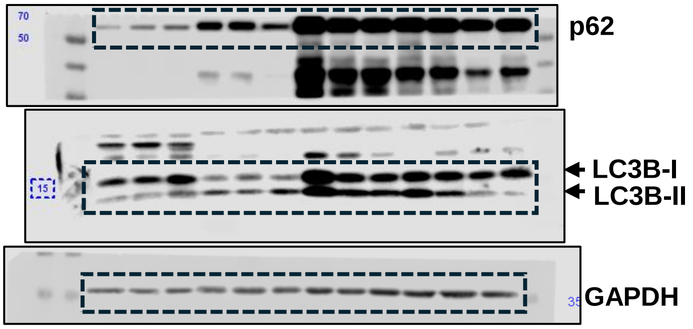

Figure 12D

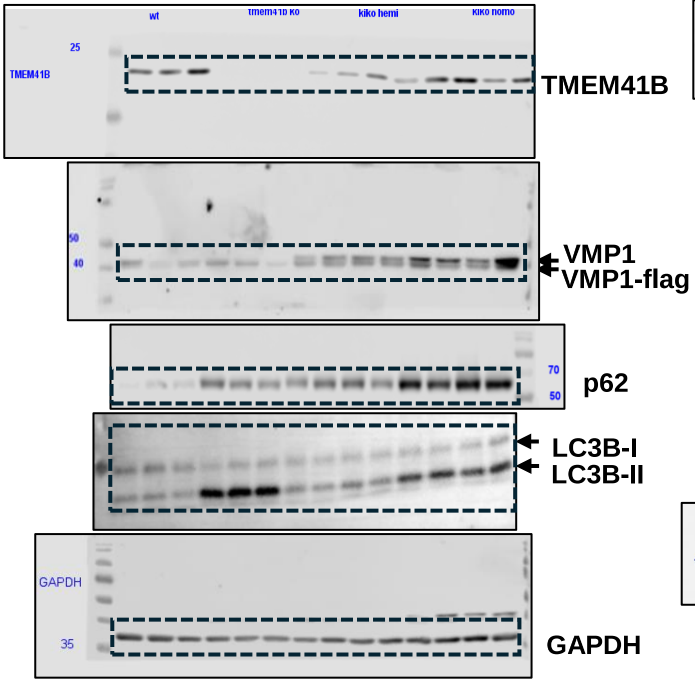

Figure 12E

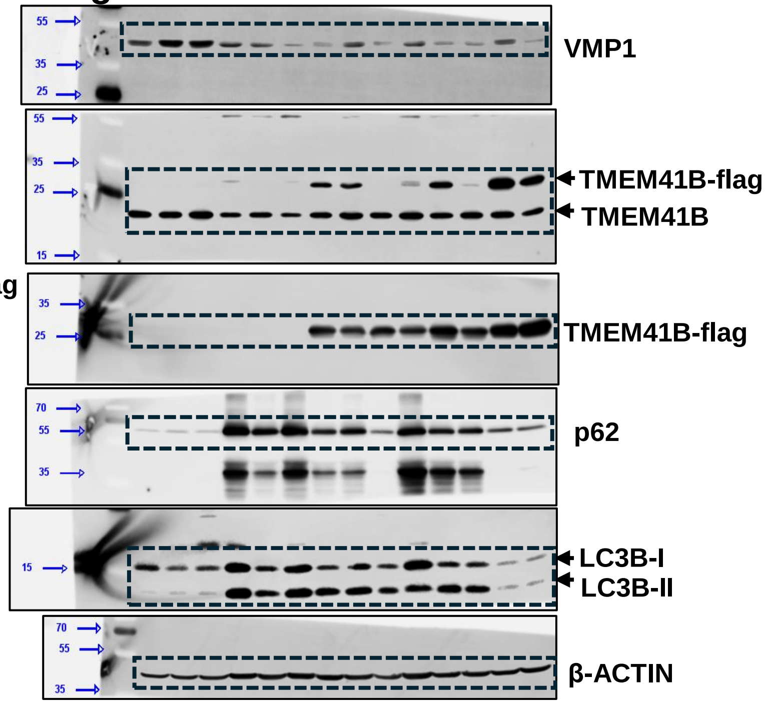

Supplement: Supplementary Material [file mmc1.pdf]
