# Supplementary material for: Overlapping Yet Distinct Functions of Vacuole Membrane Protein 1 and Transmembrane Protein 41B in Modulating Hepatic Lipoprotein Secretion and Autophagy
Source: Cell Mol Gastroenterol Hepatol. 2026 Feb 2;20(6):101751. doi: 10.1016/j.jcmgh.2026.101751 (PMC13090722; doi:10.1016/j.jcmgh.2026.101751)

cmgh
CELLULAR AND MOLECULAR GASTROENTEROLOGY AND HEPATOLOGY

ORIGINAL RESEARCH

Overlapping Yet Distinct Functions of Vacuole Membrane Protein 1 and Transmembrane Protein 41B in Modulating Hepatic Lipoprotein Secretion and Autophagy

Allen Chen,[1] Khue Nguyen,[1] Mengwei Niu,[1] Xiaoxiao Jiang,[1] Xiaochen Yu,[1] Yan Xie,[2] Wanqing Liu,[3] Nicholas O. Davidson,[2] Wen-Xing Ding,[1,4] and Hong-Min Ni[1]

[1]*Department of Pharmacology, Toxicology and Therapeutics, University of Kansas Medical Center, Kansas City, Kansas;* [2]*Division of Gastroenterology, Department of Medicine, Washington University School of Medicine, St Louis, Missouri;* [3]*Department of Pharmaceutical Sciences, Wayne State University, Detroit, Michigan; and* [4]*Department of Internal Medicine, University of Kansas Medical Center, Kansas City, Kansas*

Overlapping yet distinct functions of VMP1 and TMEM41B in modulating hepatic lipoprotein secretion and autophagy in MASH

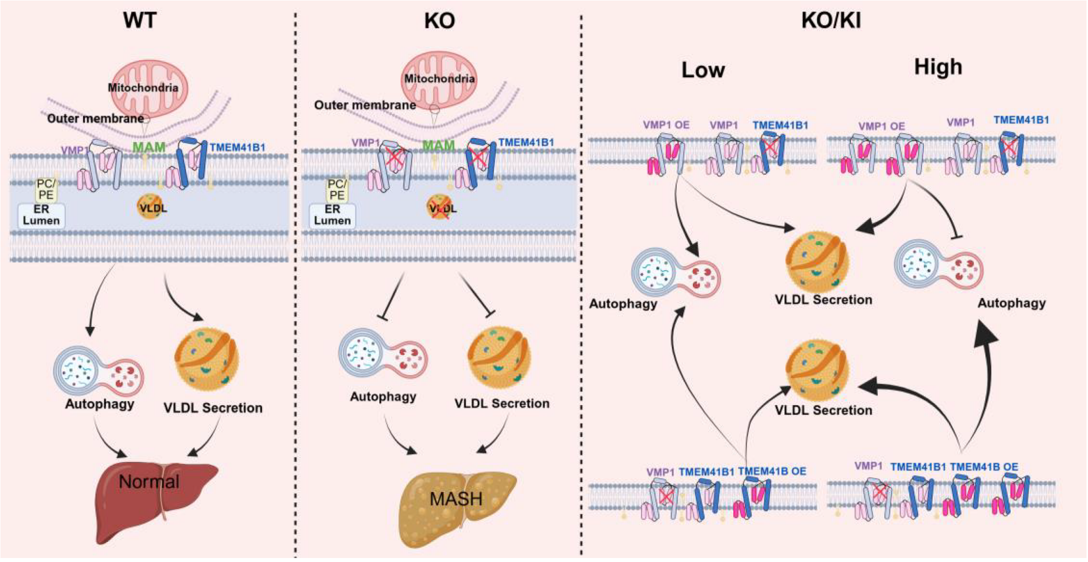

SUMMARY

Loss of hepatic vacuole membrane protein 1 or transmembrane protein 41B decreases mitochondrial-associated membrane and phospholipid content, reduces very low-density lipoprotein secretion, and promotes metabolic dysfunction-associated steatotic liver disease, via overlapping but distinct mechanisms that regulate lipoprotein secretion and autophagy. Vacuole membrane protein 1 appears to play a more critical role in regulating very low-density lipoprotein secretion and autophagy in mouse livers than transmembrane protein 41B.

WHAT YOU NEED TO KNOW

Background: Transmembrane protein 41B (TMEM41B) and vacuolar membrane protein 1 (VMP1) are endoplasmic reticulum scramblases whose roles in hepatic lipoprotein secretion and autophagy in metabolic-associated steatotic liver disease (MASLD) are unclear.

Impact: Loss of hepatic VMP1 or TMEM41B decreases VLDL secretion, leading to rapid development of MASLD through overlapping yet distinct mechanisms involving lipoprotein secretion and autophagy.

Future Directions: Whether decreased TMEM41B or VMP1 is the cause or the consequence of MASLD needs to be investigated. Targeting TMEM41B and VMP1 for the treatment of MASLD warrants further investigation.

BACKGROUND & AIMS: Transmembrane protein 41B (TMEM41B) and vacuolar membrane protein 1 (VMP1) are endoplasmic reticulum (ER) scramblases whose roles in hepatic lipoprotein secretion and autophagy in metabolic-associated steatotic liver disease (MASLD) remain unclear.

METHODS: We undertook lipidomic and functional studies in liver- and hepatocyte-specific *Tmem41b* knockout (KO) mice; *Tmem41b* knock-in (KI) mice, *Tmem41b/Vmp1* double KO (DKO); *Tmem41b* KO/*Vmp1* KI, and *Vmp1* KO/*Tmem41b* KI mice.

RESULTS: TMEM41B protein levels decreased in the livers of human subjects with MASLD. Loss of hepatic *Tmem41b* impaired very low-density lipoprotein (VLDL) secretion, with steatosis, inflammation, and fibrosis, whereas hepatic TMEM41B overexpression mitigated these effects. *Tmem41b/Vmp1* DKO mice showed further impairment in VLDL secretion compared with single *Tmem41b* KO. Lipidomic analysis revealed decreased phosphatidylcholine and phosphatidylethanolamine, with increased neutral lipids in *Tmem41b KO* mice. VMP1 and TMEM41B localize at the mitochondrial-associated membrane with reduced mitochondria-ER contact in *Vmp1* and *Tmem41b* KO mice. Loss of hepatic VMP1 or TMEM41B increased levels of LC3B-II and p62/SQSTM1, which were not further changed in DKO mice. Restoring VMP1 in *Tmem41b* KO mice and TMEM41B in *Vmp1* KO mice partially corrected defective VLDL secretion and hepatic steatosis in these single KO mice, respectively. Restoring VMP1 at a low but not a high dose corrected defective autophagy in *Tmem41b* KO mice, whereas overexpression of TMEM41B dose-dependently improved defective autophagy in *Vmp1* KO mice.

CONCLUSIONS: Loss of hepatic VMP1 or TMEM41B reduces VLDL secretion and promotes MASLD via overlapping but distinct mechanisms that regulate lipoprotein secretion and autophagy. *(Cell Mol Gastroenterol Hepatol 2026;20:101751; https://doi.org/10.1016/j.jcmgh.2026.101751)*

Keywords: Endoplasmic Reticulum; Liver Injury; MASLD; Steatosis; VLDL Secretion.

Metabolic dysfunction-associated steatotic liver disease (MASLD) has become the most common chronic liver disease globally, affecting an estimated 24% of the population and having limited United States Food and Drug Administration (FDA)-approved pharmacotherapies.[1,2] Left untreated, MASLD can progress to metabolic dysfunction-associated steatohepatitis (MASH), which is defined by hepatocyte death and inflammation in the setting of steatosis. Unaddressed, MASH can precipitate the development of end-stage liver diseases, including cirrhosis and hepatocellular carcinoma.[3,4]

Research into MASLD/MASH treatment commonly focuses on upregulating hepatic lipid catabolism or blocking absorption of lipids from the gut, thus decreasing overall systemic lipid levels.[5] Resmetirom, a liver-targeted selective thyroid hormone receptor β agonist, is the first FDA-approved drug for treating MASH. More recently, the FDA has approved semaglutide, a GLP-1 receptor agonist found in the weight-loss drugs for the treatment of patients with MASH.[6] Resmetirom works by promoting the oxidation of fatty acids in the liver, while also inhibiting de novo lipogenesis and the production of very low-density lipoproteins (VLDLs). This leads to improved lipid metabolism and reduced lipotoxicity in the liver.[7–9] Therefore, targeting hepatic lipid metabolism, including lipid synthesis (de novo), breakdown (beta oxidation), and secretion (VLDL), could potentially improve liver pathology in patients with MASLD/MASH.

VLDL secretion is a complex process. It begins in the endoplasmic reticulum (ER) membrane with the synthesis of apolipoprotein B-100 (apoB-100), the primary structural component of VLDL. Following the synthesis of apoB-100, lipids such as triglycerides (TGs) and cholesterol esters (CEs) are incorporated into the apoB-100 molecule within the ER lumen, a process facilitated by the microsomal triglyceride transfer protein (MTTP). After this lipid incorporation, VLDL is packaged into specialized transport vesicles coated with coat protein complex II (COPII) proteins and transported to the Golgi apparatus for further maturation before being secreted into the circulation.[10] Importantly, genetic variants such as patatin-like phospholipase domain-containing 3 (PNPLA3) and transmembrane 6 superfamily member 2 (TM6SF2) are associated with MASLD and both regulate the production and secretion of VLDL, highlighting the significance of VLDL secretion in MASLD.[10,11]

Vacuolar membrane protein 1 (VMP1) and transmembrane protein 41B (TMEM41B) are transmembrane

Abbreviations used in this paper: α-SMA, alpha smooth muscle actin; AAV, adeno-associated virus; ALT, alanine aminotransferase; ANOVA, analysis of variance; apoB-100, apolipoprotein B-100; BMI, body mass index; BSA, bovine serum albumin; CDAHFD, choline-deficient, amino acid-defined high-fat diet (45% fat) containing 0.1% methionine; cDNA, complementary DNA; CE, cholesteryl ester; Col1a1, collagen, type-1,alpha-1; COPII, coat protein complex II; DKO, double knockout; EM, electron microscopy; ER, endoplasmic reticulum; ES, embryonic stem; FDA, United States Food and Drug Administration; FDR, false discovery rate; FPLC, fast protein liquid chromatography; GAPDH, glyceraldehyde-3-phosphate dehydrogenase; GCKR, glucokinase regulatory protein; H&E, hematoxylin and eosin; HWT, hepatocyte wild-type; IL, interleukin; KI, knockin; KO, knockout; LD, lipid droplet; MAM, mitochondrial-associated membrane; MASLD, metabolic dysfunction-associated steatotic liver disease; MASH, metabolic dysfunction-associated steatohepatitis; MBOAT7, membrane-bound O-acyltransferase domain-containing protein 7; MS, mass spectrometry; MTTP, microsomal triglyceride transfer protein; PBS, phosphate-buffered saline; PC, phosphatidylcholine; PDI, protein disulfide isomerase; PE, phosphatidylethanolamine; PEMT, phosphatidylethanolamine N-methyltransferase; PISD, phosphatidylserine decarboxylase; PL, phospholipid; PNPLA3, patatin-like phospholipase domain-containing 3; PVDF, polyvinylidene fluoride; qRT-PCR, quantitative real-time polymerase chain reaction; RIPA, radioimmunoprecipitation; SDS, sodium dodecyl sulfate; SDS-PAGE, sodium dodecyl sulfate–polyacrylamide gel electrophoresis; SEM, standard error of the mean; TBG, thyroxine binding globulin; TG, triglyceride; TM6SF2, transmembrane 6 superfamily member 2; TMEM41B, transmembrane protein 41B; VLDL, very low-density lipoprotein; VMP1, vacuole membrane protein 1; WT, wild-type.

Most current article

© 2026 The Authors. Published by Elsevier Inc. on behalf of American Gastroenterological Association Institute. This is an open access article under the CC BY license (http://creativecommons.org/licenses/by/4.0/).
2352-345X
https://doi.org/10.1016/j.jcmgh.2026.101751

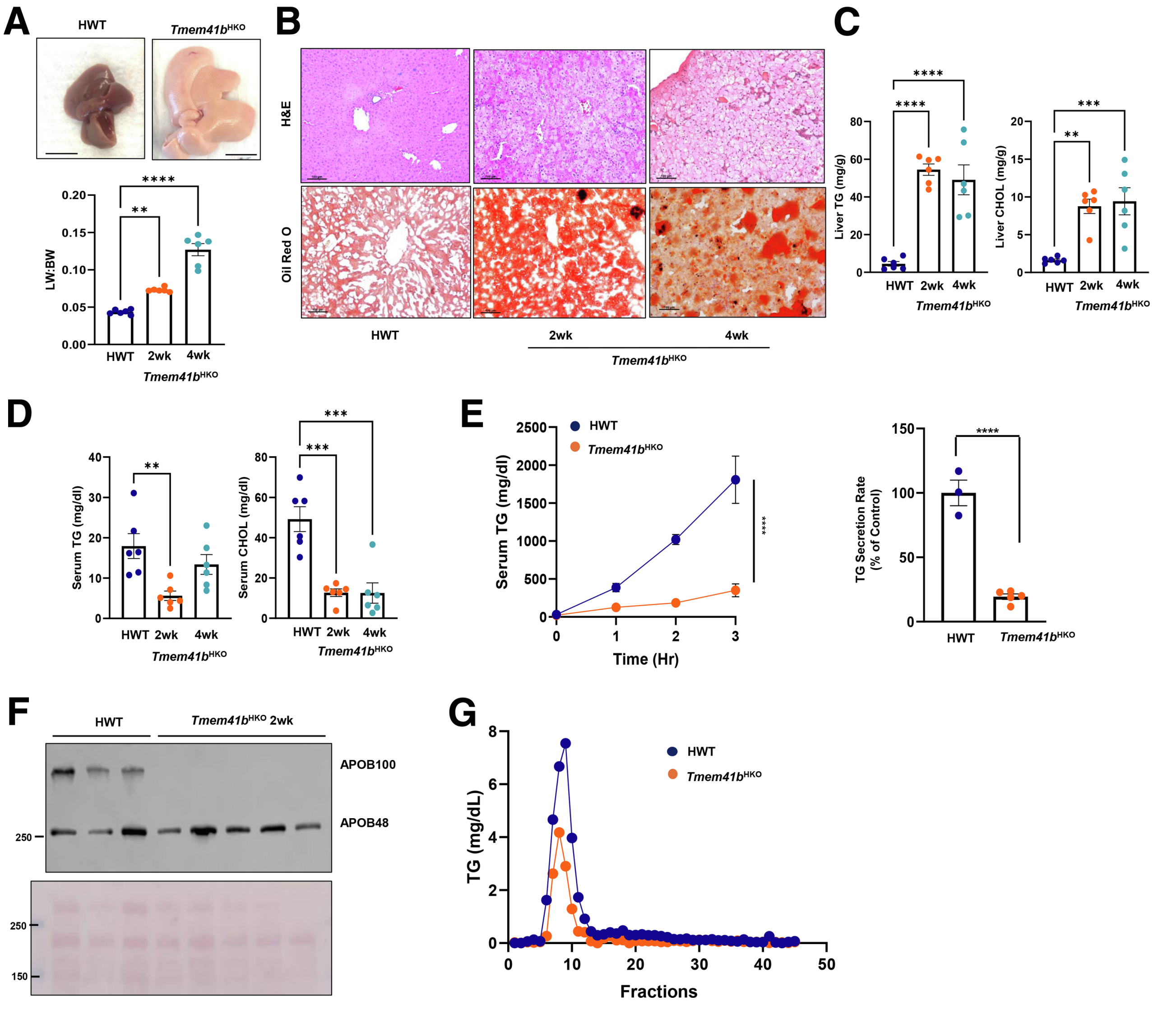

Figure 1. Hepatic deletion of TMEM41B impairs lipoprotein secretion and leads to rapid development of a microvesicular steatosis in mice. (*A*) Representative images of 8- to 12-week-old *Tmem41b*^{flox} mice at 4 weeks post AAV8-TBG-null (*Tmem41b*^{HWT}) or AAV8-TBG-cre (*Tmem41b*^{HKO}) injection with liver/body weight ratio. (*B*) H&E staining of liver tissues from *Tmem41b*^{HWT} and *Tmem41b*^{HKO} mice. Scale bars, 100 μm. Hepatic (*C*) and serum (*D*) TG and cholesterol (CHOL) were quantified. (*E*) *Tmem41b*^{flox} mice at 2 weeks post AAV injection were injected with Pluronic F-127, and serum TG concentrations were measured. TG secretion rate was quantified. (*F*) Serum APOB from *Tmem41b*^{flox} mice at 2 weeks post AAV injection were subjected to immunoblot analysis. (*G*) Mice were fasted 4 hours followed by Pluronic F-127 injection for another 3 hours. Serum from the same group of mice was pooled (N = 4). Lipoprotein profiles were analyzed by FPLC. Data represent mean ± SEM. **P* < .05; ***P* < .01; ****P* < .001; *****P* < .0001 (one-way ANOVA with post-hoc Turkey test [*A–D*] or unpaired Student's *t*-test [*E*]).

proteins located in the ER that function as phospholipid (PL) scramblases.[12–14] They play crucial roles in regulating the formation of autophagosomes and lipid droplets (LDs).[13,15–17] Recent research, including our own, has shown that VMP1 and TMEM41B are two novel regulators of hepatic VLDL secretion. Notably, both proteins are found to be decreased in the livers of mice with diet-induced MASH and in human patients with MASH.[12,16] Mice with single knockouts (KOs) of *Vmp1* and *Tmem41b* exhibit impaired VLDL secretion and rapidly develop hepatic steatosis, even when fed a standard chow diet.[12,16] However, it remains unclear whether VMP1 and TMEM41B operate through similar mechanisms or serve distinct roles in VLDL secretion and the development of MASLD. Additionally, it remains uncertain whether VMP1 and TMEM41B can compensate for each other's functions or if they operate independently or in coordination with one another in their roles regulating VLDL secretion and autophagy. In this study, we investigate the expression of TMEM41B in the livers of subjects with MASH and further explore the role of TMEM41B in relation to VMP1 regarding lipid homeostasis, autophagy, and VLDL secretion in mouse liver. Our findings offer new insights into the

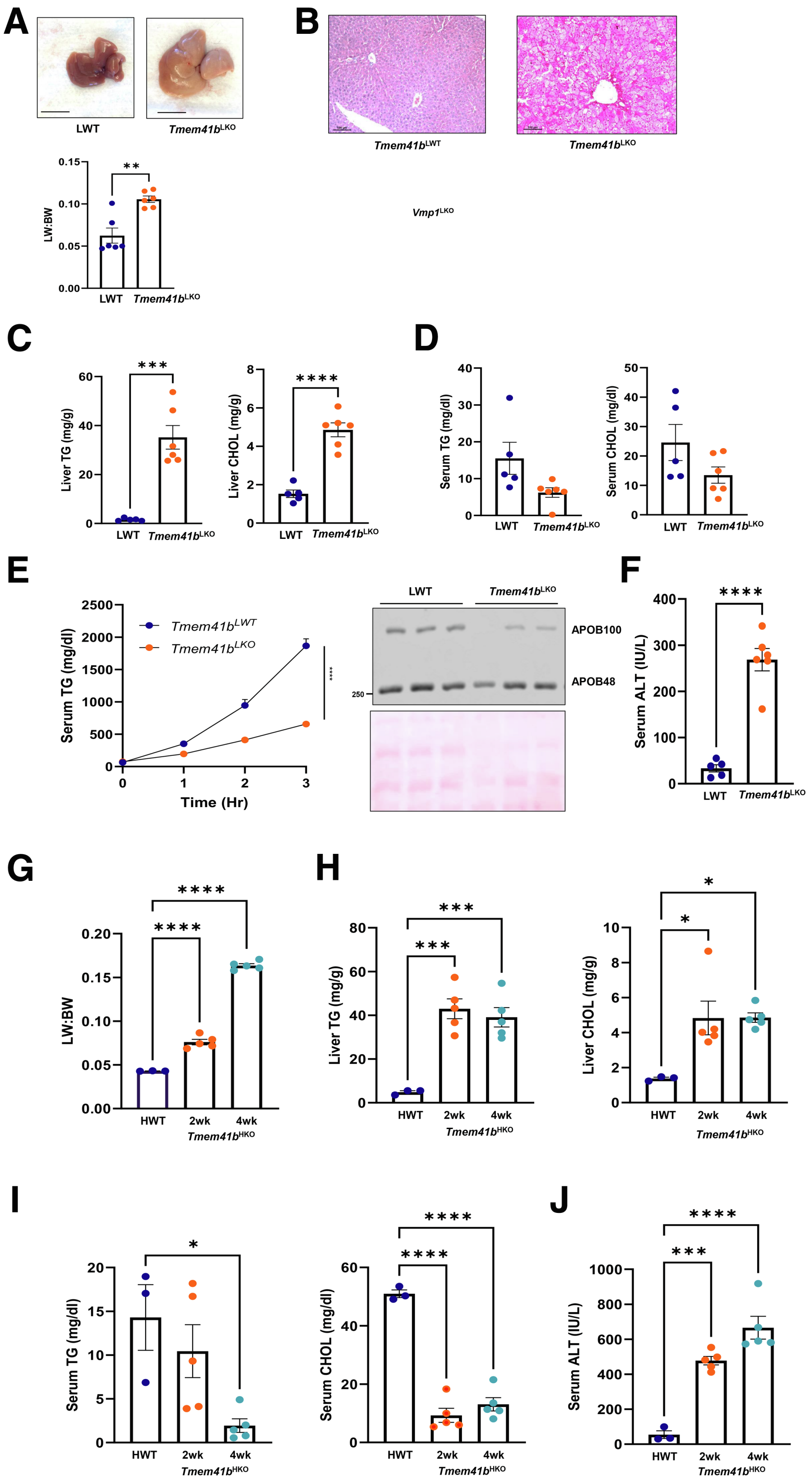

Figure 2. Hepatic deletion of TMEM41B impairs lipoprotein secretion and leads to rapid development of a microvesicular steatosis in both male and female mice. (*A*) Representative images of 1-month-old *Tmem41b*^{LKO} and matched WT mice and liver-body weight ratio. Scale bars, 1 cm. (*B*) Representative images of H&E staining of liver tissues from LWT and *Tmem41b*^{LKO} mice. Scale bars, 100 μm. Hepatic (*C*) and serum (*D*) TG and cholesterol (CHOL) were quantified. (*E*) LWT and *Tmem41b*^{LKO} mice were injected with Pluronic F-127, and serum TG concentrations were measured. Serum APOB from LWT and *Tmem41b*^{LKO} mice were subjected to immunoblot analysis. (*F*) Serum ALT activities were measured in 1-month-old *Tmem41b*^{LKO} and matched WT mice (N = 5–6). (*G*) Liver/body weight ratio in 8- to 10-week-old *Tmem41b*^{flox} female mice at 2 and 4 weeks post AAV8-TBG-cre injection. Hepatic (*H*) and serum (*I*) TG and cholesterol were measured in female mice fed ad libitum of a chow diet. (*J*) Serum ALT activities were measured in 8- to 10-week-old *Tmem41b*^{flox} female mice at 2 weeks post AAV8-TBG-cre injection (N = 3–5). Data represent mean ± SEM. *$P < .05$; **$P < .01$; ***$P < .001$; ****$P < .0001$ (unpaired Student's *t*-test [*A, C–F*] or one-way ANOVA with post-hoc Turkey test [*G–J*]).

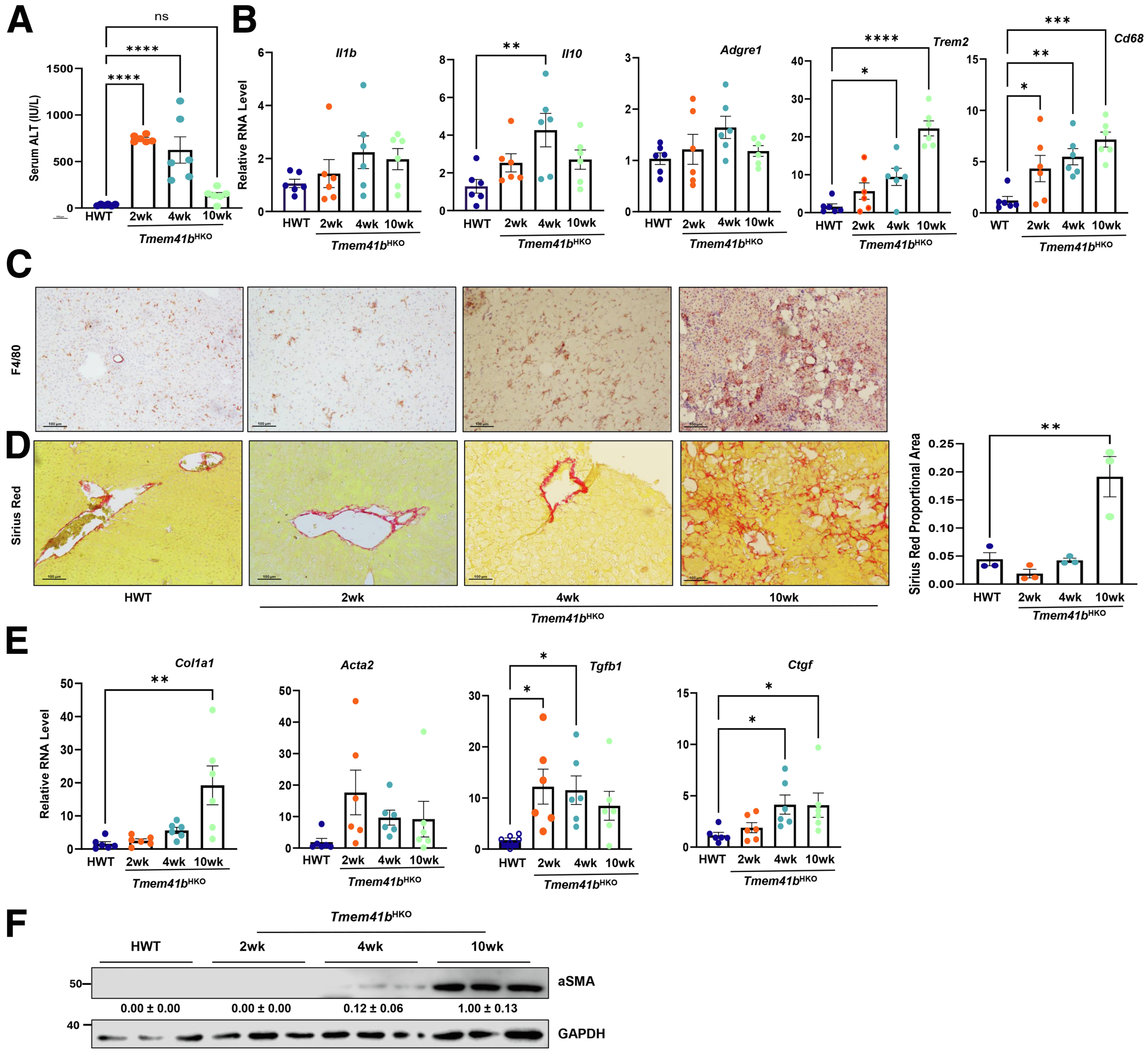

Figure 3. Hepatic deletion of TMEM41B leads to the development of MASH. (*A*) Serum ALT in *Tmem41b*^{flox} mice at 2, 4, and 10 weeks post AAV injection. (*B*) Hepatic mRNA levels of pre-inflammatory cytokines and immune cell marker genes were analyzed by qPCR and normalized to *Actb* mRNA. (*C*) Immunohistochemistry staining for F4/80 in mouse livers. (*D*) Sirius Red staining in mouse livers. (*E*) Hepatic mRNA levels of fibrogenic genes were analyzed by qPCR and normalized to *Actb* mRNA. (*F*) Immunoblot analysis of α-SMA in mouse livers. Data represent mean ± SEM. *$P < .05$; **$P < .01$; ***$P < .001$; ****$P < .0001$ (one-way ANOVA with post-hoc Turkey test).

molecular relationship between VMP1 and TMEM41B, highlighting their redundant yet distinct roles in VLDL secretion and autophagy as well as hepatic metabolism. Furthermore, we suggest that VMP1 and TMEM41B could be potential targets for treating MASLD and MASH.

Results

Hepatic Deletion of TMEM41B Impairs VLDL Secretion and Leads to Rapid Development of Microvesicular Steatosis

To investigate the physiological functions of TMEM41B in mice, 8-week-old *Tmem41b*^{flox} mice were injected with a single dose of adeno-associated virus (AAV)8- thyroxine binding globulin (TBG)-Cre (hepatocyte-specific *Tmem41b* KO, *Tmem41b*^{HKO}) or AAV8-TBG-Null (hepatocyte wild-type [HWT]) for 2 and 4 weeks. Compared with HWT mice, *Tmem41b*^{HKO} mice had enlarged and pale livers with significant increases in liver-body weight ratio (Figure 1*A*). Hematoxylin and eosin (H&E) staining revealed pan-hepatic microvesicular steatosis in *Tmem41b*^{HKO} mice (Figure 1*B*). Hepatic TG and cholesterol were significantly elevated in *Tmem41b*^{HKO} mice compared with HWT mice (Figure 1*C*). Interestingly, serum TG and cholesterol levels were significantly decreased post-AAV injection (Figure 1*D*). Therefore, we

subsequently performed lipoprotein secretion analysis in *Tmem41b*^{HKO} and HWT mice. Compared with HWT mice, serum TG was markedly decreased in *Tmem41b*^{HKO} mice in a time-dependent manner with Pluronic F-127 administration to inhibit lipolysis and tissue uptake of TG-rich lipoproteins. TG secretion rate was reduced by 81.55% in *Tmem41b*^{HKO} mice (Figure 1*E*). Immunoblot analysis revealed selectively decreased serum APOB100 in *Tmem41b*^{HKO} mice with preserved secretion of APOB48 (Figure 1*F*). Fast protein liquid chromatography (FPLC) analysis demonstrated substantially decreased VLDL-TG levels in *Tmem41b*^{HKO} mice without affecting peak distribution (Figure 1*G*), confirming impaired VLDL secretion in *Tmem41b*^{HKO} mice.

Like *Tmem41b*^{HKO} mice, 1-month-old liver-specific-*Tmem41b* KO (*Tmem41b*^{LKO}) mice also showed hepatomegaly (Figure 2*A*), massive steatosis (Figure 2*B*), markedly increased hepatic TG and cholesterol contents (Figure 2*C*), and decreased serum TG and cholesterol (Figure 2*D*). VLDL-TG and APOB100 secretion decreased in *Tmem41b*^{LKO} mice, whereas APOB48 secretion was preserved (Figure 2*E*). TG secretion rate was reduced by 66% in *Tmem41b*^{LKO} mice (Figure 2*E*). Notably, like male mice, female *Tmem41b*^{HKO} mice developed a similar increase in liver-body weight ratio, hepatic TG, and cholesterol, and a decrease in serum TG and cholesterol (Figure 2*G–I*). These data indicate that TMEM41B plays an important role in regulating hepatic APOB100-VLDL

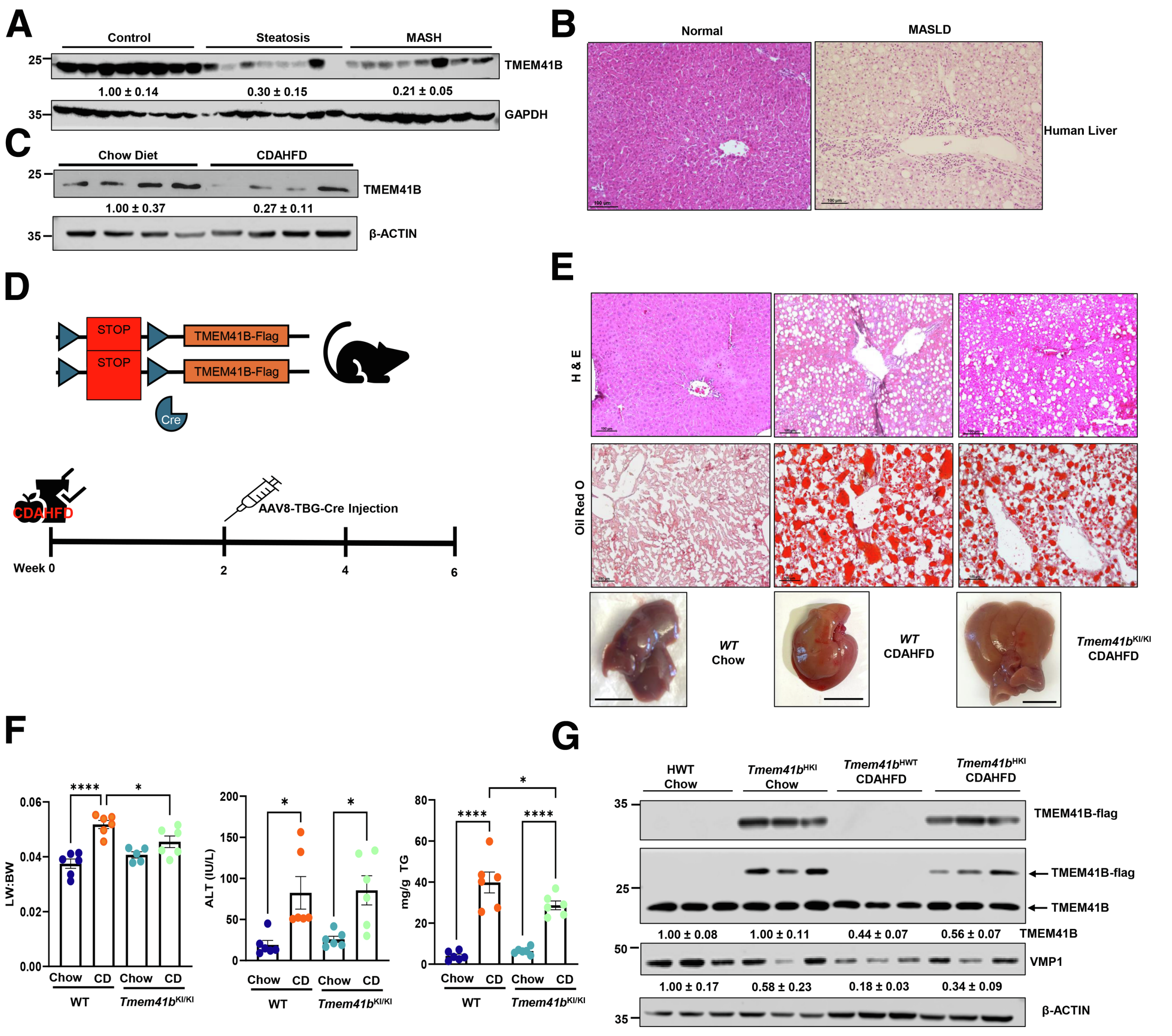

Figure 4. Reduced TMEM41B is associated with human MASLD livers and overexpression of TMEM41B alleviates diet-induced hepatic steatosis. (*A*) Immunoblot analysis of TMEM41B and (*B*) H&E staining in human livers. (*C*) Protein level of TMEM41B in mice fed with CDAHFD. (*D*) Scheme of CDAHFD-induced MASH in mice. (*E*) Representative H&E and Oil O Red images from mice. Scale bars, 50 μm. (*F*) Liver-body weight ratio, ALT, and hepatic TG in CDAHFD-fed mice. (*G*) Immunoblot analysis of TMEM41B and VMP1 in mice fed with CDAHFD. Data represent mean ± SEM. *$P < .05$; ****$P < .0001$ (one-way ANOVA with post-hoc Turkey test).

secretion and lipid homeostasis in mice, which is consistent with a previous report using guide RNA-mediated gene editing to inactivate *Tmem41b* in mouse livers.[12]

Hepatic Deletion of TMEM41B Leads to the Development of MASH

Serum levels of alanine aminotransferase (ALT) increased in *Tmem41b*^{HKO} and *Tmem41b*^{LKO} mice (Figures 2*G*, *J* and 3*A*). Hepatic mRNA levels of inflammatory genes were markedly increased in *Tmem41b*^{HKO} mice (Figure 3*B*). F4/80-positive macrophages also increased in *Tmem41b*^{HKO} mice (Figure 3*C*). *Tmem41b*^{HKO} mice displayed increased liver Sirius Red staining at 10 weeks post AAV injection (Figure 3*D*). Hepatic mRNA levels of fibrotic genes and protein levels of α-smooth muscle actin (α-SMA) were elevated in *Tmem41b*^{HKO} mice as early as 2 weeks post AAV injection compared with matched wild-type (WT) mice (Figure 3*E* and *F*). Together, these data indicate that hepatic deletion of *Tmem41b* leads to MASH in mice.

Decreased TMEM41B Is Associated With Human MASLD and Overexpression of TMEM41B Alleviates Diet-Induced Hepatic Steatosis

To examine whether TMEM41B is associated with human MASLD, we analyzed the expression of TMEM41B in livers from normal subjects and those with simple steatosis and MASH. Immunoblot analysis revealed that TMEM41B expression declined in subjects with steatosis and MASH (Figure 4*A*). H &E staining confirmed increased accumulation of LDs and immune cells in the liver of patients with MASLD (Figure 4*B*). Mice fed with choline-deficient, amino acid-defined high-fat diet (45% fat) containing 0.1% methionine (CDAHFD), a MASH diet, also exhibited decreased hepatic TMEM41B (Figure 4*C*). To examine the therapeutic effect of TMEM41B in CDAHFD-induced steatosis, conditional *Tmem41b* knockin (KI) mice were established and fed with CDAHFD (Figure 4*D*). The overexpression of TMEM41B alleviated CDAHFD-induced steatosis, as shown by improved H&E and Oil Red O staining, an enlarged liver/body weight ratio, and hepatic TG accumulation, but did not affect elevated serum ALT values. (Figure 4*E* and *F*). Overexpression of TMEM41B also restored the decreased TMEM41B caused by CDAHFD in mouse livers without affecting VMP1 expression (Figure 4*G*). These data indicate that overexpression of TMEM41B improves hepatic steatosis.

Hepatic Deletion of TMEM41B or VMP1 Leads to Decreased Hepatic Levels of Phospholipids and Disrupts ER-Mitochondria Contact

Because the deletion of *Vmp1* in the liver causes impaired VLDL secretion and MASH in mice, we proceeded to compare the effects of deleting either VMP1 or TMEM41B on changes in hepatic PLs, and the ultrastructure of the ER-mitochondria contact, as well as the ER and Golgi.[16] Unbiased lipidomic analysis revealed that the levels of phosphatidylethanolamine (PE), but not phosphatidylcholine (PC), decreased significantly in *Tmem41b*^{HKO} mice livers. However, both PC and PE decreased significantly in *Vmp1*^{HKO} mouse livers. Neutral lipids, including cholesteryl esters (CEs), diacylglycerols, and triacylglycerols, increased dramatically in *Tmem41b*^{HKO} or *Vmp1*^{HKO} mouse livers (Figure 5*A*). However, the magnitude of these changes appeared relatively more pronounced in mice lacking hepatic *Vmp1* compared with those lacking *Tmem41b*. The acyl chain length and levels of saturation of PLs play critical roles in membrane trafficking processes.[18] In the livers of *Tmem41b*^{HKO} mice, the levels of shorter acyl chain PC species decreased following AAV-TBG-Cre injection after 2 weeks, but not after 1 week. In contrast, the decrease of shorter acyl chain PC species in *Vmp1*^{HKO} mouse livers was more significant and occurred as early as 1 week post-injection. However, longer acyl chain PC species exhibited a more substantial increase in *Tmem41b*^{HKO} mouse livers compared with *Vmp1*^{HKO} mouse livers (Figure 5*B*). For PE species, both shorter and longer acyl chain species decreased in *Tmem41b*^{HKO} mouse livers post-AAV-TBG-Cre injection after 2 weeks but not after 1 week. In *Vmp1*^{HKO} mouse livers, both shorter and longer-chain PE species decreased after AAV-TBG-Cre injection at both 1 week and 2 weeks (Figure 5*B*). There were minimal changes in the hepatic metabolites of fatty acid synthesis, medium chain fatty acid, and long chain saturated and polyunsaturated fatty acid as well as various acyl carnitine fatty acid in *Tmem41b*^{HKO} mice, but these metabolites were markedly increased in *Vmp1*^{HKO} mice (Figure 6*A* and *B*). Overall, the reductions in both PC and PE species were more pronounced in *Vmp1*^{HKO} mouse livers than in *Tmem41b*^{HKO} mouse livers.

ER-mitochondria contact sites are critical for PL synthesis and calcium transport.[19] Electron microscopy (EM) analysis revealed that ER-mitochondria contact sites were readily detected in HWT, *Tmem41b*^{HKO}, and *Vmp1*^{HKO} mouse hepatocytes (Figure 7*A*). ER-mitochondria contact sites were graded based on a 4-point scale to assess the contact between ER and mitochondria, with Grade 1 being minimal contact (<25%) and Grade 4 indicating mitochondria nearly completely enveloped by ER (>75%). Quantitative EM analysis showed fewer ER-nitochondria contact sites in *Tmem41b*^{HKO} or *Vmp1*^{HKO} mouse hepatocytes than WT hepatocytes (Figure 7*B*). The loss of these contact sites seemed to be more pronounced in TMEM41B-deficient hepatocytes than in VMP1-deficient hepatocytes.

Consistent with our previous report,[16] the "LDs" observed in *Vmp1* KO hepatocytes exhibited membranes that are likely PL bilayers of the ER, facing the cytosol (indicated by white arrows). These LDs displayed clear electron-dense edges, which probably represent the surrounding PL monolayer around the lipid structures. The spaces marked by stars between the ER membrane and the electron-dense-edged lipid structures represent the ER lumen, suggesting that these LDs are stalled at the ER PL bilayer (Figure 7*C*, "#"). Interestingly, a small portion of the LDs was found within the ER lumen, which is likely

A

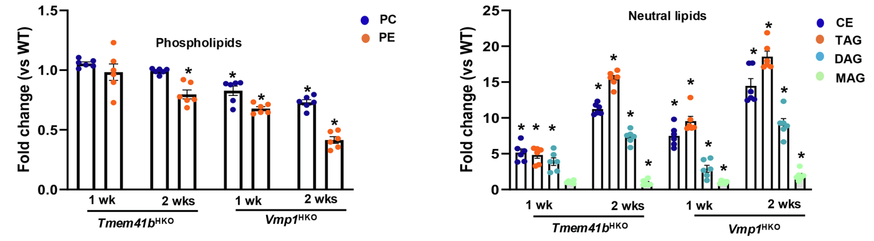

B

	Indicates the ratio of the mean scaled intensity for a metabolite between two experimental groups.
(Green)	**Green:** indicates significant difference ($P \leq .05$) between the groups shown, metabolite ratio of < 1.00
(Light green)	**Light Green:** narrowly missed statistical cutoff for significance $.05 < P < .10$, metabolite ratio of < 1.00
(Red)	**Red:** indicates significant difference ($P \leq .05$) between the groups shown; metabolite ratio of ≥ 1.00
(Light red)	**Light Red:** narrowly missed statistical cutoff for significance $.05 < P < .10$, metabolite ratio of ≥ 1.00
	**Non-colored text and cell:** mean values are not significantly different for that comparison

Pathways	Biochemical Name	Tememe41b^HKO/Tmem41b^hwt		Vmp1^HKO/Vmp1^hwt	
		1 week	2 weeks	1 week	2 weeks
Phosphatidylcholine	PC(12:0/16:1)	0.82	0.87	0.61	0.47
	PC(12:0/18:1)	0.96	0.84	0.56	0.38
	PC(12:0/18:2)	0.85	0.63	0.56	0.48
	PC(14:0/18:1)	1.02	0.77	0.54	0.50
	PC(14:0/18:2)	0.95	0.49	0.38	0.28
	PC(15:0/18:1)	0.90	0.73	0.53	0.46
	PC(15:0/18:2)	0.73	0.60	0.44	0.26
	PC(16:0/12:0)	0.88	0.64	0.62	0.36
	PC(16:0/14:0)	1.02	0.87	0.73	1.00
	PC(16:0/16:0)	0.90	0.85	0.68	0.90
	PC(16:0/16:1)	0.96	0.77	0.72	0.57
	PC(16:0/18:0)	1.05	1.05	0.78	0.79
	PC(16:0/18:1)	1.06	0.77	0.74	0.63
	PC(16:0/18:2)	0.93	0.68	0.68	0.58
	PC(16:0/20:5)	0.93	1.46	0.86	0.76
	PC(16:0/22:4)	1.16	1.43	1.19	1.04
	PC(16:0/22:5)	1.12	1.34	1.04	0.77
	PC(16:0/22:6)	1.28	1.43	1.04	0.69
	PC(17:0/14:1)	0.89	0.72	1.00	0.99
	PC(17:0/16:1)	1.01	1.27	1.00	1.00
	PC(18:1/18:1)	1.64	1.25	0.97	1.27
	PC(18:1/18:2)	1.41	1.23	1.16	1.45
	PC(18:1/18:3)	1.23	1.02	1.65	1.56
	PC(18:1/20:1)	1.29	1.07	1.07	1.31
	PC(18:1/20:2)	1.39	1.24	1.09	1.79
	PC(18:1/20:3)	1.50	1.54	1.16	1.12
	PC(18:1/20:4)	1.14	1.10	0.95	1.02
	PC(18:1/20:5)	1.22	1.83	1.45	1.53
	PC(18:1/22:4)	1.16	1.13	1.45	1.30
	PC(18:1/22:5)	1.26	1.40	1.24	0.95
	PC(18:1/22:6)	1.34	1.48	1.47	1.03
	PC(18:1/18:2)	1.41	1.23	0.88	1.18
	PC(18:1/18:3)	1.23	1.02	0.76	0.77
	PC(18:1/20:1)	1.29	1.07	1.11	1.00
	PC(18:1/20:2)	1.39	1.24	0.84	1.19
	PC(18:1/20:3)	1.50	1.54	0.93	0.87
	PC(18:1/20:4)	1.14	1.10	0.69	0.70
	PC(18:1/20:5)	1.22	1.83	0.91	0.94
	PC(18:1/22:4)	1.16	1.13	1.00	1.00
	PC(18:1/22:5)	1.26	1.40	0.89	0.94
	PC(18:1/22:6)	1.34	1.48	0.94	0.81

Pathways	Biochemical Name	Tememe41b^HKO/Tmem41b^hwt		Vmp1^HKO/Vmp1^hwt	
		1 week	2 weeks	1 week	2 weeks
Phosphotidylethanolamine (PE Ester)	PE(14:0/18:1)	1.00	1.00	0.93	0.93
	PE(14:0/18:2)	0.95	0.95	0.59	0.59
	PE(15:0/22:6)	1.08	0.90	0.68	0.48
	PE(16:0/18:2)	0.89	0.62	0.72	0.40
	PE(16:0/18:3)	0.94	0.67	0.72	0.67
	PE(16:0/20:1)	1.14	0.68	0.71	0.59
	PE(16:0/20:2)	0.80	0.58	0.61	1.06
	PE(16:0/20:3)	1.06	0.88	0.74	0.43
	PE(16:0/20:4)	1.07	0.95	0.74	0.38
	PE(16:0/20:5)	0.92	0.74	0.54	0.30
	PE(16:0/22:5)	1.13	1.07	0.80	0.50
	PE(16:0/22:6)	1.19	1.00	0.76	0.44
	PE(17:0/18:2)	0.84	0.59	0.68	0.51
	PE(17:0/20:3)	0.80	0.80	0.85	0.72
	PE(17:0/20:4)	0.87	0.63	0.67	0.37
	PE(17:0/20:5)	0.83	0.67	0.85	0.85
	PE(17:0/22:5)	0.96	0.75	0.67	0.65
	PE(17:0/22:6)	0.85	0.73	0.75	0.49

Pathways	Biochemical Name	Tememe41b^HKO/Tmem41b^hwt		Vmp1^HKO/Vmp1^hwt	
		1 week	2 weeks	1 week	2 weeks
Phosphotidylethanolamine (PE Ester)	PE(18:0/16:0)	1.07	0.95	0.72	1.23
	PE(18:0/16:1)	0.89	0.80	0.60	0.65
	PE(18:0/18:0)	0.91	0.79	0.66	0.67
	PE(18:0/18:1)	1.00	0.72	0.64	1.11
	PE(18:0/18:2)	0.80	0.56	0.66	0.41
	PE(18:0/18:3)	0.84	0.56	0.72	0.70
	PE(18:0/20:1)	0.97	0.41	0.57	0.38
	PE(18:0/20:2)	0.91	0.65	0.61	0.71
	PE(18:0/20:3)	0.98	0.79	0.76	0.48
	PE(18:0/20:4)	0.96	0.84	0.72	0.36
	PE(18:0/20:5)	0.91	0.75	0.62	0.38
	PE(18:0/22:2)	0.85	0.83	0.85	0.85
	PE(18:0/22:4)	1.11	1.01	0.86	0.88
	PE(18:0/22:5)	0.84	0.63	0.58	0.41
	PE(18:0/22:6)	0.80	0.56	0.59	0.35
	PE(18:1/16:1)	1.18	0.93	0.52	0.98
	PE(18:1/18:1)	1.63	1.00	0.66	1.61
	PE(18:1/18:2)	1.14	0.84	0.64	0.52
	PE(18:1/18:3)	1.21	0.86	0.76	0.89
	PE(18:1/20:1)	1.74	0.58	0.61	0.42
	PE(18:1/20:2)	1.53	0.88	0.58	0.76
	PE(18:1/20:3)	1.25	0.88	0.56	0.32
	PE(18:1/20:4)	1.06	0.85	0.48	0.23
	PE(18:1/20:5)	1.01	0.79	0.44	0.23
	PE(18:1/22:0)	0.98	0.77	1.00	1.00
	PE(18:1/22:1)	0.97	0.97	1.00	1.00
	PE(18:1/22:2)	0.93	0.88	0.90	0.90
	PE(18:1/22:4)	1.38	1.13	0.77	0.83
	PE(18:1/22:5)	1.21	0.80	0.51	0.44
	PE(18:1/22:6)	0.99	0.68	0.45	0.30
	PE(18:2/16:1)	0.92	0.76	0.56	0.46
	PE(18:2/18:2)	1.23	0.68	0.77	0.71
	PE(18:2/18:3)	1.00	0.66	0.55	0.60
	PE(18:2/20:1)	1.27	0.40	0.54	0.33
	PE(18:2/20:2)	1.44	0.56	0.72	0.68
	PE(18:2/20:3)	1.65	0.83	0.73	0.60
	PE(18:2/20:4)	1.00	0.77	0.56	0.43
	PE(18:2/20:5)	1.25	0.89	0.73	0.56
	PE(18:2/22:1)	0.80	0.70	0.84	0.84
	PE(18:2/22:2)	1.00	1.00	1.00	1.00
	PE(18:2/22:4)	1.28	1.02	0.99	1.00
	PE(18:2/22:5)	2.25	0.91	0.64	0.66
	PE(18:2/22:6)	1.13	0.65	0.54	0.56

Figure 5. Hepatic deletion of TMEM41B or VMP1 leads to a decline in PLs. (*A*) Total PLs and neutral lipids of mouse livers from *Tmem41b*^HKO and *Vmp1*^HKO mice by lipidomic analysis (N = 6). Data represent mean ± SEM. *$P < .05$ (unpaired Student's *t*-test). (*B*) Heatmap of PC and PE species of mouse livers from *Tmem41b*^flox or *Vmp1*^flox mice with 1 and 2 weeks post AAV8-TBG-cre injection by lipidomics analyses.

A

	Indicates the ratio of the mean scaled intensity for a metabolite between two experimental groups.
	**Green:** indicates significant difference ($P \leq .05$) between the groups shown, metabolite ratio of < 1.00
	**Light Green:** narrowly missed statistical cutoff for significance.05 < P < .10, metabolite ratio of < 1.00
	**Red:** indicates significant difference ($P \leq .05$) between the groups shown; metabolite ratio of ≥ 1.00
	**Light Red:** narrowly missed statistical cutoff for significance .05< P < .10, metabolite ratio of ≥ 1.00
	**Non-colored text and cell:** mean values are not significantly different for that comparison

Pathways	Biochemical Name	Temem41b^{HKO}/Tmem41b^{hwt}		Vmp1^{HKO}/Vmp1^{hwt}	
		1 week	2 weeks	1 week	2 weeks
Fatty Acid Synthesis	**malonylcarnitine**	**0.96**	**0.87**	0.74	0.14
	**malonate**	**1.04**	1.45	**1.02**	1.49
Medium Chain Fatty Acid	caproate (6:0)	1.03	1.22	0.66	0.74
	caprylate (8:0)	1.08	1.04	0.78	0.96
	caprate (10:0)	1.33	1.02	1.03	1.17
	5-dodecenoate (12:1n7)	0.59	0.36	1.11	1.85
Long Chain Saturated Fatty Acid	myristate (14:0)	0.76	0.91	3.16	4.29
	pentadecanoate (15:0)	0.89	1.09	1.85	2.13
	palmitate (16:0)	0.91	0.96	2.14	2.32
	margarate (17:0)	0.81	0.78	3.63	3.65
	stearate (18:0)	0.79	0.73	1.53	1.74
	nonadecanoate (19:0)	0.59	0.37	2.19	1.85
	arachidate (20:0)	1.00	0.83	4.20	2.46
Long Chain Polyunsaturated Fatty Acid (n3 and n6)	tetradecadienoate (14:2)*	0.64	0.30	0.86	1.06
	hexadecatrienoate (16:3n3)	0.81	0.48	1.36	3.46
	heptadecatrienoate (17:3)*	0.91	1.16	5.60	6.54
	stearidonate (18:4n3)	0.90	0.73	3.60	4.27
	eicosapentaenoate (EPA; 20:5n3)	0.80	0.77	2.91	2.42
	heneicosapentaenoate (21:5n3)	1.24	0.81	2.22	2.12
	docosapentaenoate (n3 DPA; 22:5n3)	1.11	0.75	2.92	2.65
	docosahexaenoate (DHA; 22:6n3)	0.91	0.54	1.36	1.38
	nisinate (24:6n3)	1.88	0.81	2.33	2.29
	hexadecadienoate (16:2n6)	1.16	1.09	4.32	6.21
	linoleate (18:2n6)	0.99	0.75	2.17	2.38
	linolenate [alpha or gamma; (18:3n3 or 6)]	0.91	0.81	3.51	4.41
	dihomo-linoleate (20:2n6)	1.30	0.89	5.47	5.15
	dihomo-linolenate (20:3n3 or n6)	1.22	0.99	2.53	2.46
	arachidonate (20:4n6)	0.75	0.68	1.32	0.97
	adrenate (22:4n6)	1.23	0.94	2.79	1.90
	docosadienoate (22:2n6)	1.29	0.75	4.71	3.20

B

Pathways	Biochemical Name	Temem41b^{HKO}/Tmem41b^{hwt}		Vmp1^{HKO}/Vmp1^{hwt}	
		1 week	2 weeks	1 week	2 weeks
Fatty Acid Metabolism (Acyl Carnitine, Short Chain)	acetylcarnitine (C2)	1.26	2.35	2.25	9.45
	valerylcarnitine (C5)	1.11	0.99	1.05	1.86
Fatty Acid Metabolism (Acyl Carnitine, Long Chain Saturated)	palmitoylcarnitine (C16)	0.84	0.85	1.04	10.26
	margaroylcarnitine (C17)*	1.17	1.15	0.59	7.40
	stearoylcarnitine (C18)	1.22	1.15	1.51	11.07
	arachidoylcarnitine (C20)*	0.91	0.44	1.04	5.72
	behenoylcarnitine (C22)*	0.77	0.41	0.72	1.35
	lignoceroylcarnitine (C24)*	0.64	0.60	1.04	1.54
Fatty Acid Metabolism (Acyl Carnitine, Monounsaturated)	palmitoleoylcarnitine (C16:1)*	1.14	1.53	2.27	11.41
	oleoylcarnitine (C18:1)	1.21	1.01	1.50	4.43
	eicosenoylcarnitine (C20:1)*	1.77	1.09	1.43	14.28
	erucoylcarnitine (C22:1)*	1.27	0.53	1.30	2.22
	nervonoylcarnitine (C24:1)*	1.07	0.65	1.14	1.44
Fatty Acid Metabolism (Acyl Carnitine, Polyunsaturated)	linoleoylcarnitine (C18:2)*	0.87	0.80	1.25	2.56
	dihomo-linoleoylcarnitine (C20:2)*	1.35	1.16	1.00	8.60
	arachidonoylcarnitine (C20:4)	1.27	1.12	1.95	3.77
	dihomo-linolenoylcarnitine (C20:3n3 or 6)*	1.85	2.42	3.24	12.69
	docosahexaenoylcarnitine (C22:6)*	0.86	1.37	1.71	2.53
Fatty Acid Metabolism (Acyl Carnitine, Hydroxy)	(R)-3-hydroxybutyrylcarnitine	1.79	0.99	0.71	0.55
	(S)-3-hydroxybutyrylcarnitine	1.99	1.41	1.45	3.40
	3-hydroxyhexanoylcarnitine (2)	1.33	1.13	0.88	0.54
	3-hydroxydecanoylcarnitine	0.98	1.41	1.81	2.24
	3-hydroxypalmitoylcarnitine	0.95	1.87	2.32	20.20
	3-hydroxyoleoylcarnitine	1.31	2.08	2.87	9.08

Figure 6. Hepatic deletion of VMP1 increases more fatty acids and acyl carnitine species than hepatic deletion of TMEM41B. Heatmap of fatty acids (*A*) and acyl carnitine species (*B*) of mouse livers from *Tmem41b*^{flox} or *Vmp1*^{flox} mice with 1 and 2 weeks post AAV8-TBG-cre injection by metabolomics analyses (N = 6).

composed of lipoproteins (denoted as "$"). Similar PL bilayer LDs were also identified in TMEM41B KO hepatocytes (Figure 7*C*, "#"). However, the number of ER luminal LDs (lipoproteins) was considerably higher in *Tmem41b* KO hepatocytes compared with *Vmp1* KO hepatocytes. Because LDs were stalled at the ER PL bilayer and lipoproteins were located inside the ER lumen, the Golgi apparatus in both *Vmp1* and *Tmem41b* KO hepatocytes was entirely devoid of lipoproteins (Figure 7*C*, *black arrows*).

To further investigate the possible effects of *Tmem41b* or *Vmp1* KO on ER-mitochondria contact, cell fractionation was performed using livers from hepatic *Tmem41b* or *Vmp1* KO mice and their WT littermates. Both TMEM41B and VMP1 were enriched at the ER and mitochondrial-associated membrane (MAM) (Figure 7*D*). Phosphatidylethanolamine N-methyltransferase (PEMT) and phosphatidylserine decarboxylase (PISD) are two enzymes important for PC and PE synthesis. Although PEMT was enriched at the ER and MAM

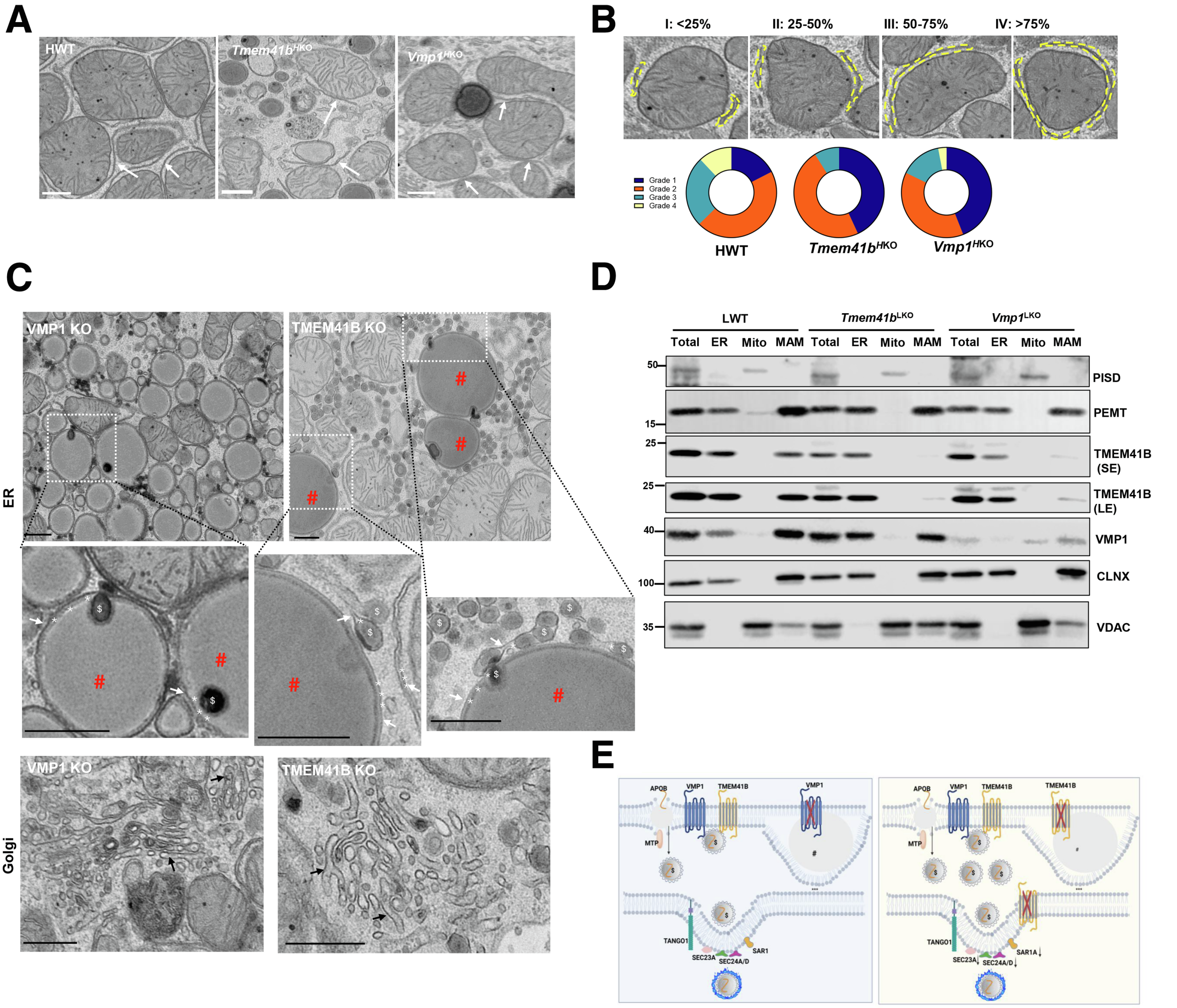

Figure 7. Hepatic deletion of *Tmem41b* or *Vmp1* disrupts ER-mitochondria contact. (*A*) Representative EM images from *Tmem41b*^{HKO} and *Vmp1*^{HKO} mice (2 weeks post AAV8 injection). *White arrows* denote close contact of ER with mitochondria approximately <30 nm, which are defined as MAM. (*B*) ER-mitochondria contact sites were quantified in mouse livers from at least 10 images. Scale bars, 500 nm. (*C*) Representative EM images of lipids from *Tmem41b*^{HKO} and *Vmp1*^{HKO} mice (2 weeks post AAV8 injection). *White arrows* denote PL bilayers of the ER. Stars denote ER lumen, # represents LDs, $ represents lipoproteins. (*D*) Subcellular fractions from 1-month-old LWT, *Tmem41b*^{LKO}, and *Vmp1*^{LKO} mice were subjected to immunoblot analysis with the indicated antibodies. (*E*) Diagrams of LDs in hepatic VMP1*Vmp1* and *Tmem41b* KO mouse hepatocytes.

with higher levels on MAM in WT mouse livers, *Tmem41b*^{LKO} or *Vmp1*^{LKO} mice had reduced total PEMT and PEMT in MAM fractions (Figure 4*E*). PISD was only located on mitochondria with no change or slight decrease in hepatic level in *Tmem41b*^{LKO} or *Vmp1*^{LKO} mice (Figure 7*D*). VMP1 protein level was reduced at MAM in *Tmem41b*^{LKO} mouse livers. Conversely, loss of VMP1 led to a profound decrease in TMEM41B localization to MAM sites. However, considerable levels of TMEM41B were detected in the ER fraction of *Tmem41b*^{LKO} mouse livers, which could be from non-parenchymal cells. To our surprise, a trace of VMP1 was observed on mitochondria fractions and needs further investigation (Figure 7*D*). Together, both VMP1 and TMEM41B localize on the MAM, and the loss of either results in decreased hepatic PL content and stalled lipid secretion at ER PL bilayer and the ER lumen (Figure 7*E*). Loss of VMP1 appears to have more profound effects on the changes of hepatic PLs and fatty acid metabolism than loss of TMEM41B.

Knockout of Both Tmem41b *and* Vmp1 *Does Not Further Exacerbate Impaired Lipoprotein Secretion, Hepatic Steatosis, and MASH Development*

To further assess the relationship between TMEM41B and VMP1 in regulating VLDL secretion, hepatic *Tmem41b* /

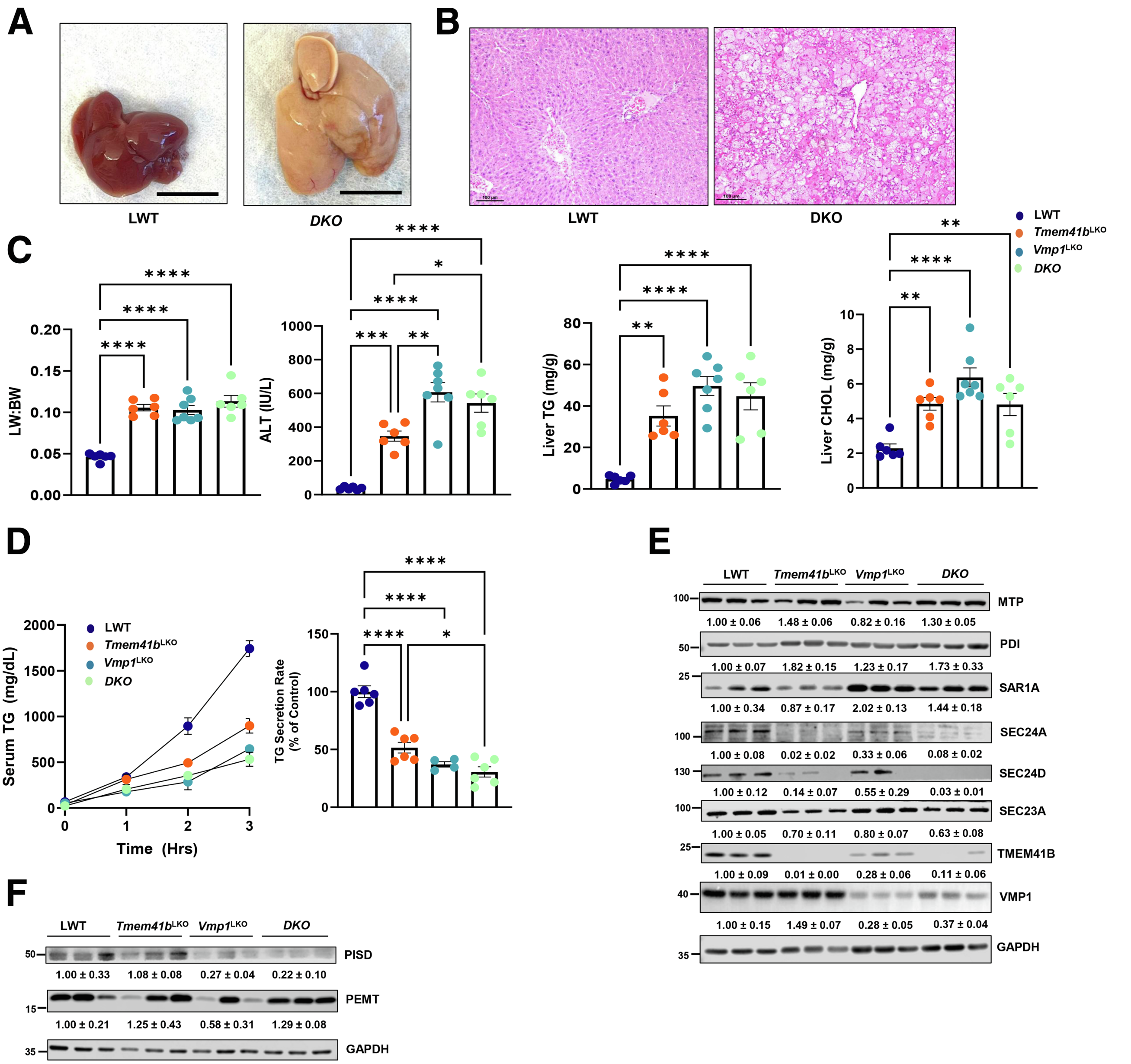

Figure 8. Knockout of both *Tmem41b* and *Vmp1* does not further exacerbate impaired lipoprotein secretion and hepatic steatosis. (*A*) Representative images of 1-month-old WT and DKO mice. Scale bars, 1 cm. (*B*) H&E staining of liver tissues from the mice. Scale bars, 100 μm. (*C*) Liver/body weight ratio, ALT, and hepatic TG and cholesterol (CHOL) were measured in mice. (*D*) LWT, *Tmem41b*^{LKO}, *Vmp1*^{LKO}, and DKO mice were injected with Pluronic F-127, and serum TG concentrations were measured. TG secretion rate was quantified. (*E*) Total liver lysates were extracted from the mice. Total mouse liver lysates were subjected to immunoblot analysis for the indicated proteins and followed by densitometric analysis. (*F*) PEMT and PISD were examined by immunoblots. Data represent mean ± SEM. **P* < .05; ***P* < .01; ****P* < .001; *****P* < .0001 (one-way ANOVA with post-hoc Turkey test).

Vmp1 double KO (DKO) mice were generated. DKO mice developed steatosis and hepatomegaly, similar to *Tmem41b*^{LKO} or *Vmp1*^{LKO} mice (Figure 8*A*–*C*). *Vmp1*^{LKO} and DKO mice had similar serum ALT levels that were higher than those of *Tmem41b*^{LKO} mice (Figure 8*C*). Hepatic TG and cholesterol increased to a similar extent in all mice (Figure 8*C*). *Vmp1*^{LKO} and DKO mice had more severe VLDL secretion defects compared with *Tmem41b*^{LKO} mice. TG secretion rate was reduced by 49.44%, 62.88%, and 69.38%, respectively, in *Tmem41b*^{LKO}, *Vmp1*^{LKO}, and DKO mice (Figure 8*D*). Expression of MTTP and disulfide isomerase (PDI), both of which are required for VLDL assembly, were increased in *Tmem41b*^{LKO} and DKO but not in *Vmp1*^{LKO} mice (Figure 8*E*). Although several COPII proteins, SEC23A, SEC24A, and SEC24D, required for exit of VLDL from ER, were moderately reduced in *Vmp1*^{LKO} mouse livers, their protein levels were decreased profoundly in either *Tmem41b*^{LKO} or *DKO* mouse livers (Figure 8*E*).

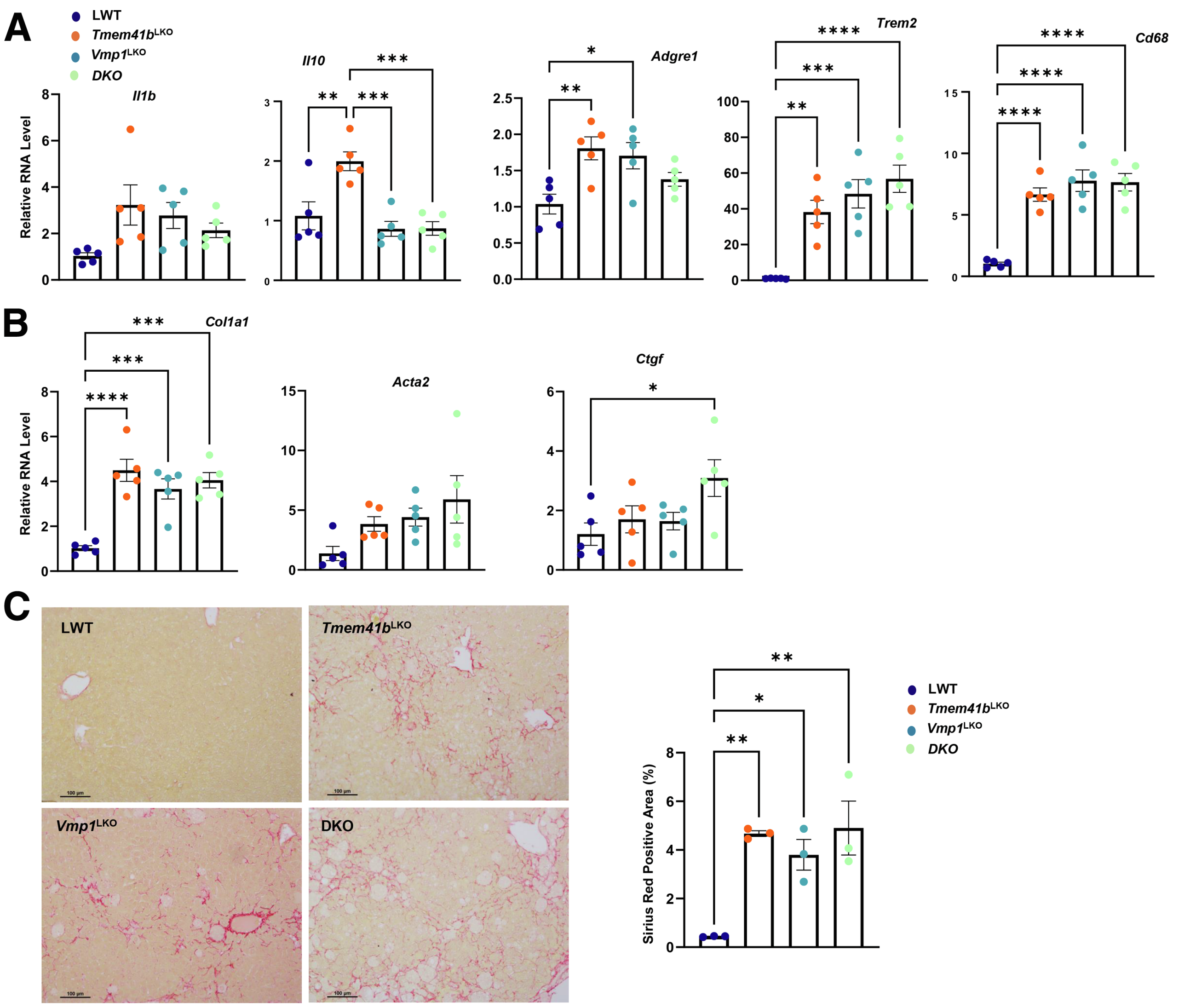

Figure 9. Knockout of both *Tmem41b* and *Vmp1* does not further exacerbate the development of MASH. (*A*) Hepatic mRNA levels of pre-inflammatory cytokines and immune cell marker genes were analyzed by qPCR and normalized to *Actb* mRNA from 1-month-old LWT, *Tmem41b*^{LKO}, *Vmp1*^{LKO}, and DKO mice (N = 5). (*B*) Hepatic mRNA levels of fibrogenic genes were analyzed by qPCR and normalized to *Actb* mRNA. (*C*) Sirius Red staining in 4-month-old mouse livers (n = 3). Data represent mean ± SEM. **P* < .05; ***P* < .01; ****P* < .001; *****P* < .0001 (one-way ANOVA with post-hoc Turkey test).

Interestingly, although loss of TMEM41B did not change VMP1 protein levels, loss of VMP1 profoundly decreased TMEM41B protein in mouse livers (Figure 8*E*). PEMT and PISD were profoundly decreased in *Vmp1*^{LKO} mouse livers and were not further decreased in DKO livers (Figure 8*F*). These results suggest that TMEM41B and VMP1 have different effects on the proteins that regulate VLDL secretion. In general, the loss of VMP1 appears to have a more significant impact, leading to greater impairment of VLDL secretion and increased liver injury.

Although *Vmp1*^{LKO} and DKO mice had higher serum ALT levels than those of *Tmem41b*^{LKO} mice (Figure 8*C*), hepatic mRNA levels of several inflammatory genes were markedly increased in all three KO mice (Figure 9*A*). Hepatic mRNA levels of fibrotic genes were elevated in all three KO mice at the similar levels compared with matched WT mice (Figure 9*B*). All KO mice developed fibrosis with increased liver Sirius Red staining (Figure 9*C*). Together, these data indicate that hepatic deletion of either *Tmem41b* or *Vmp1* leads to MASH in mice.

Overexpression of Hepatic VMP1 or TMEM41B Partially Alleviated Steatosis Caused by the Loss of the Other in Mice

To explore the relationship between VMP1 and TMEM41B in VLDL secretion, we examined whether overexpression of either VMP1 or TMEM41B could compensate for the deficiency of the other on hepatic lipid homeostasis. Overexpression of VMP1 significantly improved pale liver color in *Tmem41b*^{LKO} mice and reduced microsteatosis, as observed through H&E staining of liver sections. There was no notable difference in steatosis improvement between heterozygous and homozygous overexpression of VMP1

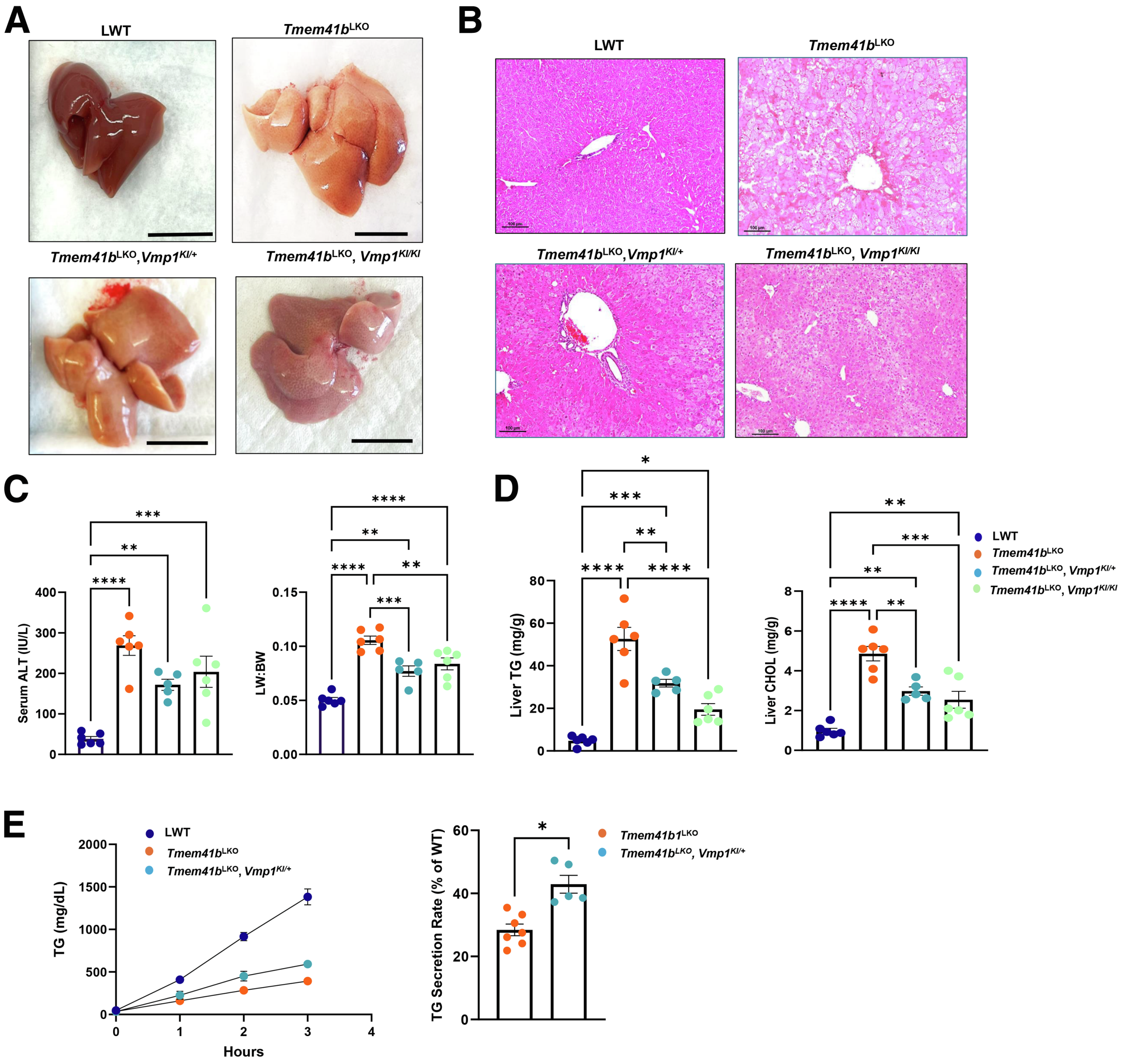

Figure 10. Overexpression of hepatic VMP1 partially alleviated steatosis caused by the loss of TMEM41B. (*A*) Representative images of 1-month-old *Tmem41b*^{LKO}, *Tmem41b*^{LKO}/*Vmp1*$^{KI/+}$, and *Tmem41b*^{LKO}/*Vmp1*$^{KI/KI}$ mice. Scale bars, 1 cm. (*B*) H&E staining of liver tissues from the mice. Scale bars, 100 μm. (*C*) Liver/body weight ratio and ALT were measured. (*D*) Hepatic TG and cholesterol (CHOL) were measured in mice. (*E*) LWT, *Tmem41b*^{LKO}, and *Tmem41b*^{LKO}/*Vmp1*$^{KI/+}$ mice were injected with Pluronic F-127, and serum TG concentrations were measured. VLDL secretion rates were quantified. Data represent mean $\pm$ SEM. *$P < .05$; **$P < .01$; ***$P < .001$; ****$P < .0001$ (one-way ANOVA with post-hoc Turkey test [*C* and *D*] and unpaired Student's *t*-test [*E*]).

(Figure 10*A* and *B*). Additionally, both heterozygous and homozygous overexpression of VMP1 partially corrected elevated levels of serum ALT and liver-to-body weight ratio in *Tmem41b*^{LKO} mice (Figure 10*C*). Overexpression of VMP1 also resulted in a significant reduction of hepatic TG and cholesterol in *Tmem41b*^{LKO} mice, regardless of whether VMP1 was overexpressed as a heterozygous or homozygous allele (Figure 10*D*). TG secretion rate was reduced in *Tmem41b*^{LKO} mice but enhanced by 14.5% with low-dose VMP1 overexpression (Figure 10*E*).

Overexpression of TMEM41B in *Vmp1*^{LKO} mice alleviated steatosis as demonstrated by H&E staining of liver sections (Figure 11*A* and *B*). Overexpression of TMEM41B also partially corrected increased liver-body weight ratio and serum levels of ALT in *Vmp1*^{LKO} mice (Figure 11*C*). Levels of hepatic TG and cholesterol were also reversed with overexpression of TMEM41B in *Vmp1*^{LKO} mice (Figure 11*D*). Notably, homozygous *Tmem41b* KI mice had much better improvement than heterozygous *Tmem41b* KI mice for the above parameters in *Vmp1*^{LKO} mice. Restoring

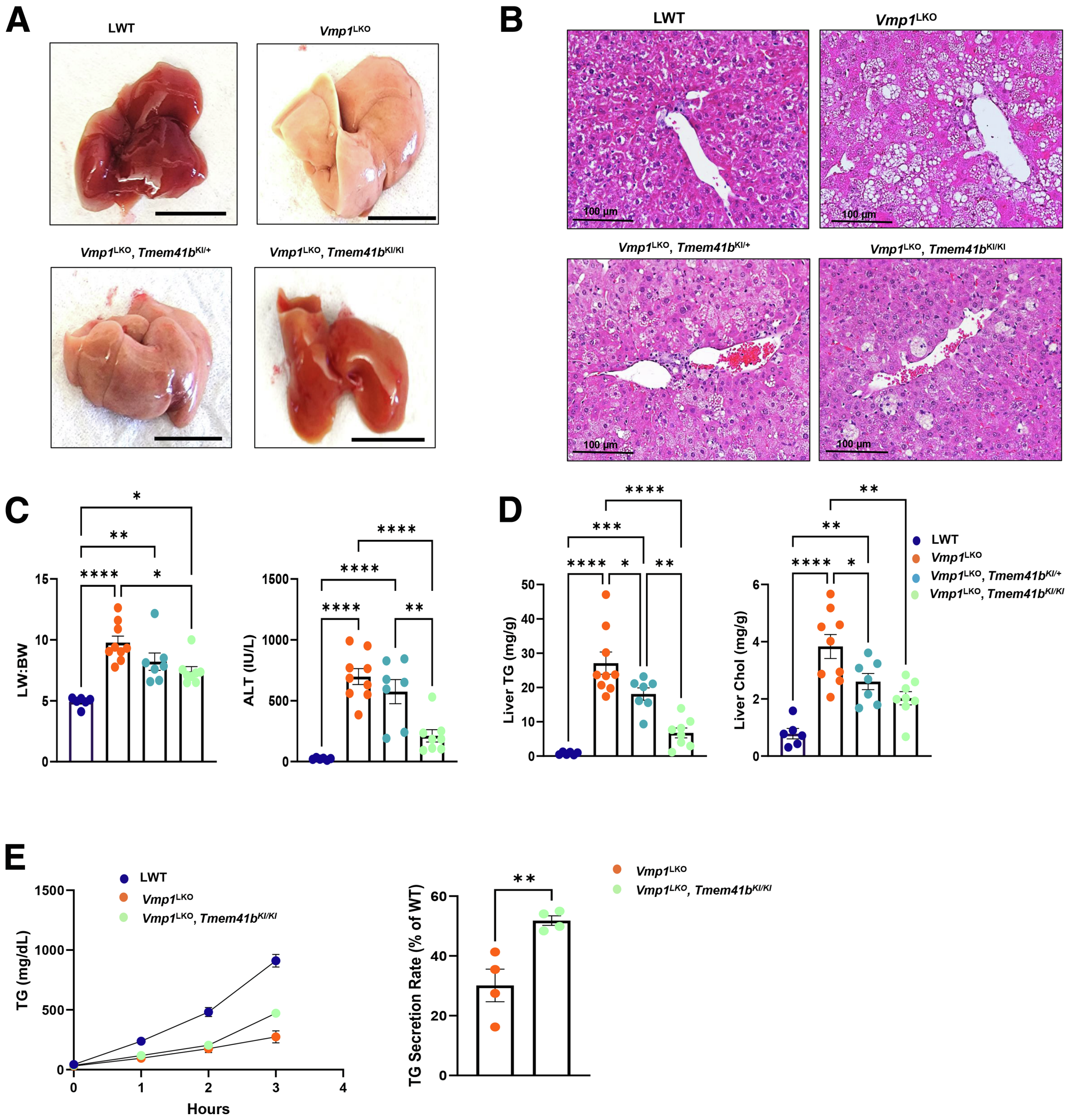

Figure 11. Overexpression of hepatic TMEM41B improved VLDL secretion and partially alleviated steatosis caused by the loss of VMP1. (*A*) Representative images of 1-month-old LWT, *Vmp1*^{LKO}, *Vmp1*^{LKO}/*Tmem41b*$^{KI/+}$, and *Vmp1*^{LKO}/*Tmem41b*$^{KI/KI}$ mice. Scale bars, 1 cm. (*B*) H&E staining of liver tissues from the mice. Scale bars, 100 μm. (*C*) Liver/body weight ratio and ALT were measured. (*D*) Hepatic TG and cholesterol (CHOL) were measured in mice. (*E*) LWT, *Vmp1*^{LKO}, and *Vmp1*^{LKO}/*Tmem41b*$^{KI/KI}$ mice were injected with Pluronic F-127, and serum TG concentrations were measured. VLDL secretion rates were quantified. Data represent mean ± SEM. **P* < .05; ***P* < .01; ****P* < .001; *****P* < .0001 (one-way ANOVA with post-hoc Turkey test [*C* and *D*] and unpaired Student's *t*-test [*E*]).

TMEM41B in *Vmp1*^{LKO} mice also improved VLDL secretion. TG secretion rate was reduced by 69.87% in *Vmp1*^{LKO} mice but enhanced by 21.7% with TMEM41B overexpression (Figure 11*E*). Together, these data indicate that VMP1 and TMEM41B functionally interact and partially compensate for the loss of the other on hepatic VLDL secretion.

Overexpression of TMEM41B in Vmp1^{LKO} *and VMP1 in* Tmem41b^{LKO} *Mice Partially Corrects Autophagy Defect*

Consistent with previous findings,[20] immunoblot analysis showed that levels of LC3B-II and p62 were increased in *Tmem41b*^{HKO} mouse livers (Figure 12*A*),

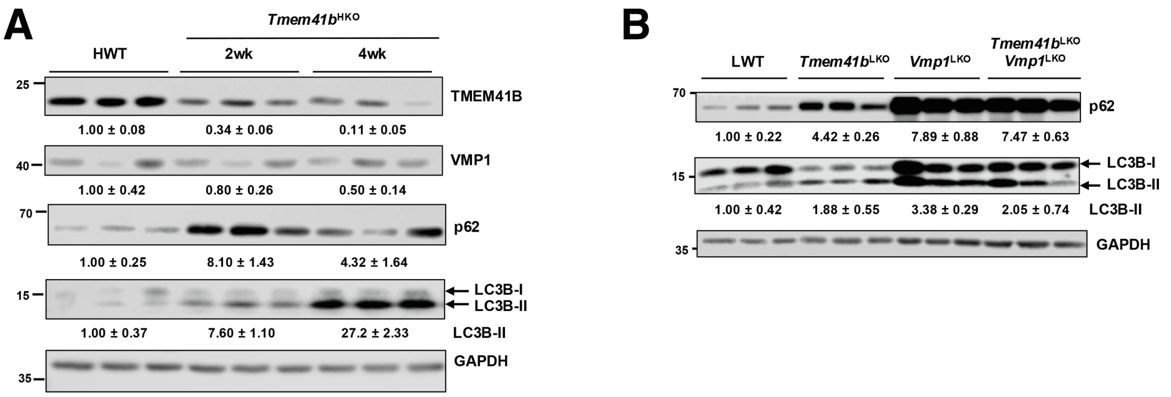

C

	Indicates the ratio of the mean scaled intensity for a metabolite between two experimental groups.
(green)	**Green:** indicates significant difference ($P \leq .05$) between the groups shown, metabolite ratio of < 1.00
(light green)	**Light Green:** narrowly missed statistical cutoff for significance $.05 < P < .10$, metabolite ratio of < 1.00
(red)	**Red:** indicates significant difference ($P \leq .05$) between the groups shown; metabolite ratio of ≥ 1.00
(light red)	**Light Red:** narrowly missed statistical cutoff for significance $.05 < P < .10$, metabolite ratio of ≥ 1.00
	**Non-colored text and cell:** mean values are not significantly different for that comparison

Pathways	Biochemical Name	Tmem41b^{HKO}/Tmem41b^{hwt} 1 week	Tmem41b^{HKO}/Tmem41b^{hwt} 2 weeks	Vmp1^{HKO}/Vmp1^{hwt} 1 week	Vmp1^{HKO}/Vmp1^{hwt} 2 weeks
Dipeptide	alanylleucine	1.07	0.43	0.52	0.45
	glycylisoleucine	0.80	0.18	0.38	0.32
	glycylleucine	0.72	0.22	0.47	0.10
	glycylvaline	0.70	0.30	0.47	0.11
	histidylalanine	1.00	1.00	0.62	0.32
	isoleucylglycine	0.97	0.33	0.40	0.05
	leucylglycine	0.97	0.45	0.72	0.22
	phenylalanylalanine	1.40	0.62	0.71	0.19
	phenylalanylglycine	1.00	0.26	0.57	0.12
	prolylglycine	0.66	0.44	0.51	0.18
	tyrosylglycine	1.13	0.29	0.36	0.06
	valylglycine	0.96	0.29	0.50	0.04

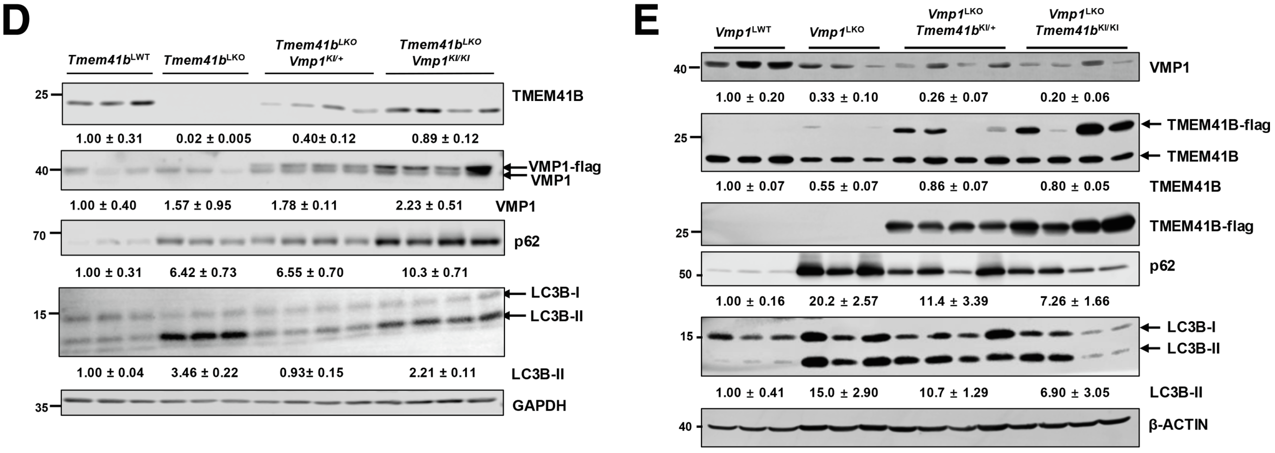

indicating impaired autophagy by deletion of *Tmem41b* in mouse livers. The levels of LC3-II and p62 were comparable in *Vmp1*^{LKO} *and DKO* mice but were much higher than those of *Tmem41b*^{LKO} mice (Figure 12*B*), suggesting VMP1 may be more critical than TMEM41B in regulating autophagy in mouse livers. Consistent with this, the levels of hepatic dipeptides generated by autophagic lysosomal degradation decreased in *Tmem41b*^{LKO} and *Vmp1*^{LKO} mice, with a more pronounced decrease observed in *Vmp1*^{LKO} mice compared with *Tmem41b*^{LKO} mice (Figure 12*C*). We next examined whether overexpression of either VMP1 or TMEM41B could compensate for the deficiency of the other on hepatic autophagy in mouse livers. Heterozygous overexpression of VMP1 completely corrected increased levels of hepatic LC3-II but not p62 in *Tmem41b*^{LKO} mouse livers (Figure 12*D*). Homozygous overexpression of VMP1 further increased LC3B-II and p62 compared with heterozygous overexpression of VMP1 (Figure 12*E*). Levels of LC3B-II and p62 were markedly increased in *Vmp1*^{LKO} mouse livers compared with matched WT mouse livers. Heterozygous overexpression of TMEM41B partially corrected increased levels of hepatic LC3-II and p62 in *Vmp1*^{LKO} mouse livers, which were further corrected by homozygous overexpression of TMEM41B (Figure 12*F*). These results suggest that loss of VMP1 has more profound defects on hepatic autophagy than loss of TMEM41B. TMEM41B and VMP1 may partially compensate for the loss of the other in hepatic autophagy; however, a precise level of VMP1 is critical for effective autophagy in hepatocytes.

Discussion

In this study, we found that *Vmp1*^{LKO} mice developed more severe VLDL secretion defects and liver injury than *Tmem41b*^{LKO} mice, and that hepatic deletion of both *Vmp1* and *Tmem41b* did not further impair VLDL secretion and worsen liver injury compared with *Vmp1*^{LKO} mice. *Vmp1*-deficient hepatocytes exhibited increased ER bilayer "LDs" and fewer ER luminal "LDs," whereas *Tmem41b*-deficient hepatocytes showed increased ER bilayer "LDs" and a greater number of ER luminal "LDs." Overexpression of *Vmp1* or *Tmem41b* in *Tmem41b*-deficient or *Vmp1*-deficient mouse livers partially alleviated steatosis and corrected autophagy defects, suggesting TMEM41B and VMP1 functionally interact and compensate for hepatic VLDL secretion and autophagy. However, the restoration of VLDL secretion and hepatic autophagy by *Tmem41b* in *Vmp1*-deficient mouse livers occurs in a gene-dose-dependent manner. In contrast, overexpression of either low or high levels of *Vmp1* in *Tmem41b*-deficient mouse livers partially improved VLDL secretion and hepatic steatosis, but high levels of *Vmp1* fail to rescue hepatic autophagy defects, implying that VMP1 and TMEM41B have similar yet distinct roles in regulating hepatic VLDL secretion and autophagy. Previous studies comparing predicted TMEM41B and VMP1 structures have identified sequence homology between the two proteins. Notably, both VMP1 and TMEM41B contain a DedA domain consisting of two transmembrane helices, one extramembrane helix, and two reentrant loops. TMEM41B and VMP1 seem to structurally differ primarily in their N-termini, in which TMEM41B contains fewer transmembrane domains, but an extramembrane domain is not present in VMP1.[21,22] Whether these structural differences contribute to differences in their regulation of hepatic VLDL secretion, liver injury, and autophagy remains to be investigated.

Conventional LDs are cytosolic organelles that store metabolic energy and consist of a hydrophobic core of neutral lipids surrounded by a PL monolayer decorated with proteins. Histological analysis with H&E staining of liver sections revealed a significant increase in the accumulation of hepatic LDs in both *Tmem41b*-deficient and *Vmp1*-deficient mouse livers. EM analysis of the ultrastructure of these LDs in *Vmp1* KO hepatocytes showed that the large LDs appear to be stalled within the ER membrane bilayer, consistent with our previous findings.[16] Additionally, we observed some small LDs inside the ER lumen, surrounded by ER membranes. Interestingly, we also identified hybrid LDs, where large ER membrane bilayer LDs coexist with smaller luminal LDs in the same ER structure. These ER luminal LDs are more prominent in *Tmem41b* KO hepatocytes compared with *Vmp1* KO hepatocytes. These small ER luminal LDs likely represent lipoproteins transported from the ER bilayer into the ER lumen for further lipidation and maturation. A previous study, which utilized a gRNA against *Tmem41b* and AAV-TBG-Cre injection in mice harboring the lox-STOP-lox Cas9 cassette, demonstrated that TMEM41B-deficient hepatocytes lack mature lipoproteins with increased LDs encapsulated by highly curved ER membranes.[12] These data suggest that TMEM41B and VMP1 are necessary for VLDL biogenesis in the ER.

How do TMEM41B and VLDL regulate VLDL biogenesis, and what differences exist between them? Three processes critical for VLDL secretion occur at specific ER sites. These include: (1) import of neutral lipids from the ER bilayer

Figure 12. (*See previous page*). **Overexpression of TMEM41B in *Vmp1*^{LKO} and VMP1 in *Tmem41b*^{LKO} mice partially corrects autophagy defect.** (*A*) Total lysates were extracted from *Tmem41b*^{flox} mice at 2 and 4 weeks post AAV injection and subjected to the immunoblot analyses with the indicated antibodies, followed by densitometric analysis. (*B*) Total lysates were extracted from LWT, *Tmem41b*^{LKO}, *Vmp1*^{LKO}, and DKO mice and subjected to immunoblot analyses with the indicated antibodies, followed by densitometric analysis. (*C*) Heatmap of dipeptide of mouse livers from *Tmem41b* ^{flox} or *Vmp1* ^{flox} mice with 1 and 2 weeks post AAV8-TBG-cre injection by metabolomics analyses (N = 6). (*D*) Total lysates were extracted from LWT, *Tmem41b*^{LKO}, *Tmem41b*^{LKO}/*Vmp1*$^{KI/+}$, and *Tmem41b*^{LKO}/*Vmp1*$^{KI/KI}$ mice and subjected to the immunoblot analyses with the indicated antibodies, followed by densitometric analysis. (*E*) Total lysates were extracted from LWT, *Vmp1*^{LKO}, *Vmp1*^{LKO}/*Tmem41b*$^{KI/+}$, and *Vmp1*^{LKO}/*Tmem41b*$^{KI/KI}$ mice and subjected to the immunoblot analyses with the indicated antibody, followed by densitometric analysis.

into the ER lumen; (2) assembly of pre-VLDL and maturation in the ER lumen; and (3) export of pre-VLDL from the ER lumen. ER membrane PL content and composition are critical for ER membrane remodeling, curvature, and tension, which are also crucial for LD budding from the ER bilayer.[21,23] The amount of PC and PE, as well as the fatty acyl chain compositions of PC and PE, especially PC and PE arachidonyl chain, has been shown to regulate VLDL secretion.[18,24] The decreased hepatic PC and PE content, together with the changes to the acyl-chain composition of PC and PE in *Tmem41b* or *Vmp1* KO hepatocytes may thus change the biophysical tension and curvature of the ER membrane, arresting import of neutral lipids from the ER membrane bilayer to the ER lumen. *Vmp1* KO hepatocytes have a more profound decrease of total PL and short- and long-chain PC and PE species than *Tmem41b* KO hepatocytes, which may explain the greater number of LDs stalled in the ER membrane bilayer than *Tmem41b* KO hepatocytes. MAM is critical in regulating PL synthesis and transfer between the ER and mitochondria. TMEM41B KO and *Vmp1* KO hepatocytes have decreased ER-mitochondria contact sites. Furthermore, both TMEM41B and VMP1 are located at MAM, and PEMT and PISD, two necessary PL synthesis enzymes, were reduced in *Tmem41b* and *Vmp1* KO livers, which may contribute to reduced PL synthesis. Although TMEM41B and VMP1 are both ER scramblases that regulate the PL distribution between the outer and inner leaflet of the ER membrane,[13,15,25] it remains unclear whether TMEM41B and VMP1 would have different scramblase activity. Both TMEM41B and VMP1 possess a DedA domain and were structurally predicted to serve as half transporters for ion and lipid transport.[26] Future work to dissect the structural differences of these two similar proteins may yield further mechanistic insights to better understand how they regulate the ER membrane dynamics and VLDL biogenesis.

Inside the ER lumen, it is known that MTTP is required to transfer the bulk of triglycerides into APOB100 for VLDL assembly. Deletion of MTTP abolishes VLDL secretion. MTTP activity is enhanced by PDI, which is regulated by the IRE1α-XBP1s-PDI axis, and decreased IRE1α-mediated PDI production impairs hepatic VLDL secretion in mice.[27–29] Pre-VLDL particles are progressively lipidated, which requires MTTP and other players, including TM6SF2.[30] Liver-specific *Tm6sf2* KO mice exhibit steatosis and reduced VLDL secretion with small, underlipidated VLDL particles without affecting APOB48 secretion. We did not find significant changes in either hepatic MTTP or PDI in *Tmem41b* or *Vmp1* KO hepatocytes, suggesting that alterations in MTTP and PDI are less likely to contribute to steatosis in these KO mice. Assembly of pre-VLDL in the ER lumen is APOB-dependent.[31] It has been reported that TMEM41B and VMP1 directly interact with APOB100.[12,32] We found that hepatic APOB100 levels markedly decreased in hepatic *Tmem41b* or *Vmp1* KO mouse serum, which may indicate preferential impacts on pre-VLDL assembly in the ER lumen impairing VLDL secretion.[33]

At the ER exit site, it is known that TANGO1 and TALI are critical in regulating the export of the pre-VLDL from the ER. Moreover, the COPII-coated vesicle is assembled through a signaling pathway beginning with SAR1 and SURF4 and progressing through SEC proteins at the ER exit site to facilitate delivering pre-VLDL to the Golgi for further processing.[34] It has been reported that TMEM41B interacts with SURF4 at ER exit sites to assist in COPII-coated vesicle formation.[12] VMP1 also interacted with SEC24D and substantially colocalized with SEC24D in hepatocytes of mouse livers. TMEM41B and VMP1 may likely increase the stability of APOB and COPII complex proteins.[16] However, more profound decreases of SAR1A, SEC24A, SEC24D, and SEC23A were found in *Tmem41b* KO mouse livers than *Vmp1* KO mouse livers, suggesting that TMEM41B seems to be a more important regulator of COPII-coated vesicle transport at the ER exit sites than VMP1. Future studies are needed to dissect how TMEM41B and VMP1 might regulate hepatic APOB and COPII proteins at either post-translational, transcriptional, or both. Notably, it has been reported that VMP1 and TMEM41B interact with each other.[20] As deletion of VMP1 decreased TMEM41B levels, TMEM41B and VMP1 potentially stabilize each other within hepatocytes.

In addition to their function in VLDL secretion, TMEM41B and VMP1 also regulate autophagy at an early step, likely by promoting autophagosome closure.[20,35,36] Similar to a previous report,[20] we found the autophagy defect to be more severe in *Vmp1*^{LKO} mouse livers than in *Tmem41b*^{LKO} livers. Hepatic deletion of both TMEM41B and VMP1 did not further exacerbate impaired autophagy. We also found that low-level VMP1 overexpression partially corrected autophagy defect in *Vmp1*-deficient mouse livers. However, high-level VMP1 overexpression impaired autophagy in *Tmem41b*-deficient mouse livers, suggesting that too much VMP1 may also impair autophagy, although the mechanisms behind this hormesis dose response need further study. TMEM41B could correct autophagy defects in *Vmp1*^{LKO} mouse livers in a dose-dependent manner, unlike a previous report using cultured HEK293 cells.[20] Likely, the fine-tuned TMEM41B and VMP1 levels in mouse hepatocytes in vivo could be different from those of cultured HEK293 cells. Notably, several in vitro studies showed that VMP1 deficiency causes an increase in membrane contact sites such as ER-mitochondria, ER-endosomes, ER-LDs, and ER-autophagosomes through VAP-mediated or other mechanisms, suggesting that VMP1 inhibits contact site formation in HeLa, COS7, and MEF cells.[15,36,37] In contrast to VMP1, the involvement of TMEM41B in membrane contact has not been well-investigated. We and others found that ER-mitochondria contact sites or perhaps total ER were reduced in *Tmem41b*- or *Vmp1*-deficient hepatocytes.[12,16] It seems that organelle contact regulation by VMP1 and TMEM41B may be cell-type dependent. However, how TMEM41B and VMP1 regulate ER-mitochondria and other organelle contact sites, along with their roles in regulating autophagy, needs further investigation.

A recent report identified rare and low-frequency ATG7 loss-of-function variants that promote MASLD progression by impairing autophagy and facilitating ballooning and inflammation in the European population.[38] Increased cell

death, inflammation, and fibrosis have been observed in hepatic *Atg5* and *Atg7-deficient* mice, which have impaired autophagy, but not impaired VLDL secretion and steatosis.[39,40] However, most genetically modified mouse MASLD models, such as hepatic *Surf4-, Lpcat3-, Mea6-,* and *Sar1b*-deficiency mice, showed defective hepatic VLDL secretion and increased hepatic steatosis, though none of them developed MASH.[18,24,34,41–43] Therefore, the MASH phenotypes in VMP1 and TMEM41B KO mice are distinct from those of MASLD models. The MASH phenotypes in VMP1 and TMEM41B KO mice are likely due to combined impaired VLDL secretion and autophagy, which is unique to TMEM41B and VMP1.

Although obesity is a common risk factor for MASLD, about 10% to 20% of MASLD patients worldwide have a normal body mass index (BMI) or are lean, a condition called lean-MASLD. Genetic factors linked to lean-MASLD include variants in PNPLA3, TM6SF2, glucokinase regulatory protein (GCKR), and membrane-bound O-acyltransferase domain-containing protein 7 (MBOAT7), which affect lipid metabolism and hepatic lipid accumulation.[44,45] Notably, mutations in PNPLA3 and TM6SF2 can disrupt VLDL secretion. Additionally, carrying rare APOB variants is associated with advanced MASLD, reflecting impairments in VLDL secretion.[46,47] Although mutations or polymorphisms in TMEM41B and VMP1 have not yet been identified in humans with MASH, lower levels of these genes in mice fed a MASLD diet and in human MASH liver samples suggest that downregulation of TMEM41B and VMP1 is important in human MASLD.

In summary, we found that human MASH livers have decreased hepatic VMP1 and TMEM41B. Our results indicate that the lack of hepatic TMEM41B and VMP1 impairs VLDL secretion and autophagy, resulting in MASH in mice. Overexpression of VMP1 alleviates steatosis but not autophagy at high expression levels, whereas overexpression of TMEM41B improves VLDL secretion, ameliorates steatosis, and partially corrects autophagy defects. TMEM41B and VMP1 likely have both redundant and distinct roles in regulating VLDL secretion and autophagy, and future studies designed to boost hepatic TMEM41B but not VMP1 may be beneficial in enhancing VLDL secretion and improving MASH pathology in humans.

Materials and Methods

Animals

The *Vmp1^{flox}* and *Vmp1* conditional KI mice were described previously.[16] The *Tmem41b^{flox}* and *Tmem41b* conditional KI mice were generated in collaboration with Cyagen. Briefly, to generate *Tmem41b^{flox}* mice, embryonic stem (ES) cells containing *Tmem41b* exons 3–5 cassette which were flanked by 2 loxP sequences. Cre-mediated depletion of exons 3–5 leads to a frameshift, resulting in a small truncated peptide. To generate *Tmem41b* conditional KI mice, ES cells containing an insertion of *Tmem41b-3×Flag* cassette, which contains a polyA tail flanked by 2 *loxP* sequences between CAG promoter and *Tmem41b-3×Flag* under *Rosa26* loci were injected into C57BL/6N blastocysts to obtain chimeric mice. The chimeric mice were crossed with C57BL/6N to obtain mutant mice. Cre-mediated depletion of the polyA tail leads to expression of TMEM41b-FLAG. *Tmem41b^{flox,} Vmp1^{flox}* mice were generated by crossing *Tmem41b^{flox}* with *Vmp1^{flox}* mice. *Tmem41b ^{flox}*, *Vmp1*^{KI} mice were generated by crossing *Tmem41b*^{flox} mice with *Vmp1*^{KI} mice. *Vmp1^{flox}*, *Tmem41b^{KI}* mice were generated by crossing *Vmp1^{flox}* mice with *Tmem41b^{KI}* mice. To generate liver-specific KO/KI mice, the mutant mice were crossed with albumin-Cre mice. To generate hepatocyte-specific *Tmem41b* (H-*Tmem41b)* KO mice, *Tmem41b^{flox}* were injected intravenously with AAV8-TBG-null or AAV8-TBG-cre (1×10^{11} GC/mouse). All mice were fed with a chow diet unless otherwise indicated. To generate a model of diet-induced MASH, mice were fed a CDAHFD (#A6071309, Research Diet) for 6 weeks. Mice were specific pathogen-free and maintained in a barrier rodent facility under standard experimental conditions. The Institutional Animal Care and Use Committee of the University of Kansas Medical Center approved all procedures.

Reagents and Antibodies

Antibodies and reagents used in this study are listed in Table 1.

Hepatic TG and Cholesterol Analysis

Hepatic lipid extraction was performed as described previously.[48] Briefly, frozen liver tissues (20–50 mg) were homogenized and followed by lipid extraction using a chloroform-methanol mixture. The resulting lipid extracts were analyzed for TG and cholesterol content using the GPO-Triglyceride Reagent Set and Cholesterol liquid reagent (catalog #T7532 and #C7510, Pointe Scientific), following the manufacturer's protocols.

In Vivo VLDL Secretion Assay

In vivo VLDL secretion assay was performed as previously described.[49] Mice were subjected to a 4-hour fast prior to the intraperitoneal administration of Pluronic F-127 at a dosage of 10 mg/kg. Pluronic F-127 inhibits both the lipolysis and the tissue uptake of lipoproteins in mice. Blood samples were collected at baseline (pre-injection) and subsequently at hourly intervals for a duration of 3 hours post-injection. TG concentrations were quantified using the colorimetric assay described above.

Blood Biochemistry Analysis

Serum TG and cholesterol levels were quantified using the colorimetric assays outlined above. Serum ALT activity was assessed using commercially available kit (catalog #A7526, Pointe Scientific) in accordance with the manufacturer's instructions.

Histology and Immunohistochemistry Analysis

Livers were preserved in 10% neutral buffered formalin and subsequently embedded in paraffin. Sections of 5-μm thickness from the paraffin blocks were stained using H&E

Table 1. List of Antibodies and Reagents Used in the Study

Name	Citation	Supplier	Cat no.	Clone no.
Antibodies				
Anti-APOB (rabbit polyclonal)	PMID: 33445170	Washington U. (Dr Nicholas Davidson)		
Anti-Calnexin (rabbit polyclonal)		Santa Cruz Biotechnology	sc-11397	H-70
Anti-F4/80 (rat monoclonal)		Invitrogen	14-4801-85	BM8
Anti-FLAG (mouse monoclonal)		Sigma-Aldrich	F3165	M2
Anti-GAPDH (rabbit monoclonal)		Cell Signaling Technology	2118	14C10
Anti-LC3 (rabbit polyclonal)	PMID: 27151180			
Anti-MTP (mouse monoclonal)		Santa Cruz Biotechnology	sc-135994	8
Anti-p62 (mouse monoclonal)		Abnova	H00008878	M01
Anti-PEMT (rabbit polyclonal)		ThermoFisher	PIPA542383	
Anti-PDI (rabbit polyclonal)		Sigma-Aldrich	P7372	MD-12
Anti-PISD (rabbit polyclonal)		Proteintech	16401-1-AP	
Anti-SAR1A (rabbit polyclonal)		Proteintech	15350-1-AP	
Anti-SEC23A (rabbit polyclonal)		Cell Signaling Technology	8162	
Anti-SEC24C (rabbit polyclonal)		Cell Signaling Technology	8531	
Anti-SEC24D (rabbit monoclonal)		Cell Signaling Technology	14687	D9M7L
Anti-TMEM41B (rabbit polyclonal)		Proteintech	29270-1-AP	
Anti-TMEM41B (rabbit polyclonal)		Sigma-Aldrich	HPA014946	
Anti-VMP1 (rabbit monoclonal)		Cell Signaling Technology	12929	D1Y3E
Anti-α-SMA (rabbit polyclonal)		Abcam	ab5694	
Anti-β-actin (mouse monoclonal)		Sigma-Aldrich	A5441	AC-15
Anti-VDAC (rabbit polyclonal)		Calbiochem	AB10527	
HRP-goat anti-rabbit		Jackson Immuno Research	111-035-144	
HRP-goat anti-mouse		Jackson Immuno Research	115-035-146	
HRP-goat anti-rat		Jackson Immuno Research	112-035-143	
HRP-goat anti-rat		Vector Laboratories	MP-7444	
Others				
AAV8-TBG-iCre		Vector Biolabs		
AAV8-TBG-null		Vector Biolabs		
ALT (SGPT) liquid reagents		Pointe Scientific	A7526	
CDAHFD		Research Diet	A6071309	
Cholesterol liquid reagents		Pointe Scientific	C7510	
Oil red O		Sigma-Aldrich	O0625	
Pluronic F-127		Thermo Fisher	p6866	
Ponceau S		Sigma-Aldrich	P3504	
Protease inhibitor cocktail		Bimake	B14002	
2xSYBR Green qPCR Master Mix		Bimake	B21202	
Triglycerides liquid reagents		Pointe Scientific	T7532	
TRIzol reagent		ThermoFisher Scientific	15596026	
HiMark^{TM} prestained protein standard		ThermoFisher Scientific	LC5699	
PageRuler^{TM} prestained protein ladder		ThermoFisher Scientific	PI26617	

for histopathological assessment. For immunohistochemical analysis, F4/80 was utilized to identify macrophages, whereas Sirius Red staining was employed to visualize collagen deposition.

Oil Red O Staining

Oil Red O staining was performed using liver cryosections as described previously.[50] Livers were preserved in 4% paraformaldehyde overnight at 4°C, then infiltrated with 20% sucrose overnight at the same temperature, and embedded in Tissue-Tek OCT Compound. Sections of 6-μm thickness were stained with Oil Red O in 60% isopropanol for 15 minutes at 37°C. This was followed by a wash with 60% isopropanol and 3 washes with water, then stained with hematoxylin, concluding with a final wash with water.

EM Analysis

EM was performed as described previously.[51] Briefly, livers were perfused with 2.5% glutaraldehyde in a 0.1 M sodium cacodylate buffer and subsequently sectioned into small fragments. Ultra-thin sections were then stained with uranyl acetate and lead citrate. Imaging was performed using a JEM 1016CX electron microscope (JEOL) to analyze the ultrastructural details.

Immunoblot Analysis

Liver proteins were extracted using radioimmunoprecipitation (RIPA) buffer, containing 1% NP40, 0.5% sodium deoxycholate, and 0.1% sodium dodecyl sulfate (SDS) in phosphate-buffered saline (PBS). A total of 30 μg of protein was separated on a sodium dodecyl sulfate–polyacrylamide gel electrophoresis (SDS-PAGE) gel, followed by transfer onto a polyvinylidene fluoride (PVDF) membrane. The membranes were subsequently incubated with appropriate primary antibodies, followed by corresponding secondary antibodies, and detection was performed using SuperSignal Plus chemiluminescent substrate (Thermo Fisher Scientific). Densitometric

Table 2. Lists of Primer Sequences

Name	Forward (5′–3′)	Reverse (5′–3′)
Acta2	CCACCGCAAATGCTTCTAAGT	GGCAGGAATGATTTGGAAAGG
Actb	TGTTACCAACTGGGACGACA	GGGGTGTTGAAGGTCTCAAA
Adgre	CTTTGGCTATGGGCTTCCAGTC	GCAAGGAGGACAGAGTTTATCGTG
Cd68	TGCGGCTCCCTGTGTGT	TCTTCCTCTGTTCCTTGGGCTAT
Col1a1	TGTGTTCCCTACTCAGCCGTCT	CATCGGTCATGCTCTCTCCAA
Ctgf	CTGCCAGTGGAGTTCAAATGC	TCATTGTCCCCAGGACAGTTG
Il1b	GCCCATCCTCTGTGACTCAT	AGGCCACAGGTATTTTGTCG
Il10	CCAAGCCTTATCGGAAATGA	TTTTCACAGGGGAGAAATCG
Tgfb1	TGCTAATGGTGGACCGCAA	CACTGCTTCCCGAATGTCTGA
Trem2	TATGACGCCTTGAAGCACTG	AGAGTGATGGTGACGGTTCC

analysis of the bands was conducted using ImageJ or Un-Scan-It software, with normalization to β-actin or glyceraldehyde-3-phosphate dehydrogenase (GAPDH). All densitometry data are reported as mean ± standard error of the mean (SEM).

Quantitative Real-time Polymerase Chain Reaction

RNA was extracted from murine hepatic tissue using Trizol reagent (Thermo Fisher Scientific) and subsequently reverse transcribed into complementary DNA (cDNA) utilizing RevertAid H minus reverse transcriptase (Thermo Fisher Scientific).[52] Quantitative real-time polymerase chain reaction (qRT-PCR) was conducted on a Bio-Rad CFX384 detection system employing SYBR Green mix (Bimake). Expression of *Acta2, Adgre, Cd68, Col1a1, Ctgf, Il1b, Il10, Tgfb1, and Trem2* was quantified using qRT-PCR analysis, and *Actb* was used as an internal control. The fold change of mRNA was expressed as $2^{-\Delta\Delta Ct}$. All the primer sequences used in this study are listed in Table 2.

Lipidomics and Metabolomics Analysis

Lipidomic and metabolomic profiling of mouse liver tissues was conducted at Metabolon, Inc as described previously.[16,53] Briefly, for lipidomic analysis, lipids were extracted from samples in a methanol:dichloromethane mixture in the presence of internal standards. After concentration under nitrogen, the lipid extracts were reconstituted in 0.25 mL of 10 mM ammonium acetate dichloromethane:methanol (50:50). These extracts were subsequently transferred to inserts and loaded into vials for infusion mass spectrometry (MS) analysis, utilizing a Shimadzu LC equipped with nano PEEK tubing and the Sciex SelexION-5500 QTRAP. The analysis was conducted in both positive and negative ionization modes. The 5500 QTRAP operated in multiple reaction monitoring (MRM) mode, with over 1100 monitored reactions. Quantification of individual lipid species was achieved by calculating the peak area ratios of the target lipids to their corresponding internal standards, which were then normalized to the known concentrations of the internal standards added during extraction. Lipid class concentrations were derived as the aggregate of all molecular species within each class, whereas fatty acid profiles were delineated by determining the constituency of each class based on individual fatty acids. Statistical evaluations were conducted using ArrayStudio, R (http://cran.r-project.org/), or JMP, with significance assessed via 1-way analysis of variance (ANOVA). The false discovery rate (FDR) was estimated utilizing q-values to adjust for multiple comparisons.

Metabolomics analysis was performed by Metabolon as described previously.[53] Following log transformation and imputation of missing values, if any, with the minimum observed value for each compound, Welch's 2-sample *t*-test was used to identify biochemicals that differed significantly between experimental groups. A summary of the numbers of biochemicals that achieved statistical significance ($P \leq .05$), as well as those approaching significance ($.05 < P < .10$) is shown.

Subcellular Fractionation

Subcellular fractionation was performed using an adapted, previously described protocol.[54] Mouse livers from 1-month-old LWT, *Tmem41b*^{LKO} and *Vmp1*^{LKO} mice were homogenized in a mannitol-sucrose-bovine serum albumin (BSA)-EGTA-Tris buffer using a Potter-Elvehjem tissue homogenizer. Mitochondria were separated from cytosolic fractions through a series of 9000 g to 10,000 g centrifugations. Following this, 95,000 g to 100,000 g centrifugations in 30% vol/vol Percoll solution was performed to separate MAM from mitochondria. ER was isolated from cytosolic fractions after subjecting the supernatant from a 20,000 g centrifugation to a 100,000 g centrifugation then collecting pellet. Subcellular fractions were subsequently performed using immunoblot analysis.

Statistical Analysis

Data were analyzed in R or with GraphPad. All experimental data are expressed as mean ± SEM and subjected to unpaired Student's *t*-test (2-group comparisons) or 1-way ANOVA with post-hoc Turkey test (multigroup comparisons).

Supplementary Material

Note: To access the supplementary material accompanying this article, visit the full text version at https://doi.org/10.1016/j.jcmgh.2026.101743.

References

1. Tincopa MA, Anstee QM, Loomba R. New and emerging treatments for metabolic dysfunction-associated steatohepatitis. Cell Metab 2024;36:912–926.
2. Younossi ZM, Blissett D, Blissett R, et al. The economic and clinical burden of nonalcoholic fatty liver disease in the United States and Europe. Hepatology 2016;64:1577–1586.
3. Israelsen M, Francque S, Tsochatzis EA, Krag A. Steatotic liver disease. Lancet 2024;404:1761–1778.
4. Wang X, Zhang L, Dong B. Molecular mechanisms in MASLD/MASH-related HCC. Hepatology 2025;82:1303–1324.
5. Ipsen DH, Lykkesfeldt J, Tveden-Nyborg P. Molecular mechanisms of hepatic lipid accumulation in non-alcoholic fatty liver disease. Cell Mol Life Sci 2018;75:3313–3327.
6. Sanyal AJ, Newsome PN, Kliers I, et al. Phase 3 trial of semaglutide in metabolic dysfunction-associated steatohepatitis. N Engl J Med 2025;392:2089–2099.
7. Harrison SA, Bashir MR, Guy CD, et al. Resmetirom (MGL-3196) for the treatment of non-alcoholic steatohepatitis: a multicentre, randomised, double-blind, placebo-controlled, phase 2 trial. Lancet 2019;394:2012–2024.
8. Harrison SA, Taub R, Neff GW, et al. Resmetirom for nonalcoholic fatty liver disease: a randomized, double-blind, placebo-controlled phase 3 trial. Nat Med 2023;29:2919–2928.
9. Polyzos SA, Targher G. Hepatic thyroid hormone receptor-beta signalling: mechanisms and recent advancements in the treatment of metabolic dysfunction-associated steatohepatitis. Diabetes Obes Metab 2025;27:1635–1647.
10. van Zwol W, van de Sluis B, Ginsberg HN, Kuivenhoven JA. VLDL biogenesis and secretion: it takes a village. Circ Res 2024;134:226–244.
11. Trepo E, Valenti L. Update on NAFLD genetics: from new variants to the clinic. J Hepatol 2020;72:1196–1209.
12. Huang D, Xu B, Liu L, et al. TMEM41B acts as an ER scramblase required for lipoprotein biogenesis and lipid homeostasis. Cell Metab 2021;33:1655–1670.e1658.
13. Li YE, Wang Y, Du X, et al. TMEM41B and VMP1 are scramblases and regulate the distribution of cholesterol and phosphatidylserine. J Cell Biol 2021;220:e202103105.
14. Chen A, Zhang C, Ding WX, Ni HM. Mechanisms of scramblases in regulating hepatic lipoprotein secretion and autophagy. J Lipid Res 2026;67:100979.
15. Reinisch KM, Chen XW, Melia TJ. "VTT"-domain proteins VMP1 and TMEM41B function in lipid homeostasis globally and locally as ER scramblases. Contact (Thousand Oaks) 2021;4:25152564211024494.
16. Jiang X, Fulte S, Deng F, et al. Lack of VMP1 impairs hepatic lipoprotein secretion and promotes non-alcoholic steatohepatitis. J Hepatol 2022;77:619–631.
17. Morita K, Hama Y, Mizushima N. TMEM41B functions with VMP1 in autophagosome formation. Autophagy 2019;15:922–923.
18. Rong X, Wang B, Dunham MM, et al. Lpcat3-dependent production of arachidonoyl phospholipids is a key determinant of triglyceride secretion. Elife 2015;4:e06557.
19. Voeltz GK, Sawyer EM, Hajnoczky G, Prinz WA. Making the connection: How membrane contact sites have changed our view of organelle biology. Cell 2024;187:257–270.
20. Morita K, Hama Y, Izume T, et al. Genome-wide CRISPR screen identifies TMEM41B as a gene required for autophagosome formation. J Cell Biol 2018;217:3817–3828.
21. Ben M'barek K, Ajjaji D, Chorlay A, et al. ER membrane phospholipids and surface tension control cellular lipid droplet formation. Dev Cell 2017;41:591–604.e597.
22. Hama Y, Morishita H, Mizushima N. Regulation of ER-derived membrane dynamics by the DedA domain-containing proteins VMP1 and TMEM41B. EMBO Rep 2022;23:e53894.
23. Fei W, Shui G, Zhang Y, et al. A role for phosphatidic acid in the formation of "supersized" lipid droplets. PLoS Genet 2011;7:e1002201.
24. Hashidate-Yoshida T, Harayama T, Hishikawa D, et al. Fatty acid remodeling by LPCAT3 enriches arachidonate in phospholipid membranes and regulates triglyceride transport. Elife 2015;4:e06328.
25. Chen A, Ding WX, Ni HM. Scramblases as regulators of autophagy and lipid homeostasis: implications for NAFLD. Autophagy Rep 2022;1:143–160.
26. Okawa F, Hama Y, Zhang S, et al. Evolution and insights into the structure and function of the DedA superfamily containing TMEM41B and VMP1. J Cell Sci 2021;134:jcs255877.
27. Wang S, Chen Z, Lam V, et al. IRE1alpha-XBP1s induces PDI expression to increase MTP activity for hepatic VLDL assembly and lipid homeostasis. Cell Metab 2012;16:473–486.
28. Newberry EP, Strout GW, Fitzpatrick JAJ, Davidson NO. Liver-specific deletion of Mttp versus Tm6sf2 reveals distinct defects in stepwise VLDL assembly. J Lipid Res 2021;62:100080.
29. Raabe M, Veniant MM, Sullivan MA, et al. Analysis of the role of microsomal triglyceride transfer protein in the liver of tissue-specific knockout mice. J Clin Invest 1999;103:1287–1298.
30. Newberry EP, Hall Z, Xie Y, et al. Liver-specific deletion of mouse Tm6sf2 promotes steatosis, fibrosis, and hepatocellular cancer. Hepatology 2021;74:1203–1219.
31. Davidson NO, Shelness GS. APOLIPOPROTEIN B: mRNA editing, lipoprotein assembly, and presecretory degradation. Annu Rev Nutr 2000;20:169–193.
32. Morishita H, Zhao YG, Tamura N, et al. A critical role of VMP1 in lipoprotein secretion. Elife 2019;8:e48834.
33. Kulinski A, Rustaeus S, Vance JE. Microsomal triacylglycerol transfer protein is required for lumenal accretion of triacylglycerol not associated with ApoB, as

well as for ApoB lipidation. J Biol Chem 2002;277:31516–31525.
34. Santos AJ, Nogueira C, Ortega-Bellido M, Malhotra V. TANGO1 and Mia2/cTAGE5 (TALI) cooperate to export bulky pre-chylomicrons/VLDLs from the endoplasmic reticulum. J Cell Biol 2016;213:343–354.
35. Ding WX, Ma X, Kim S, et al. Recent insights about autophagy in pancreatitis. eGastroenterology 2024;2:e100057.
36. Zhao YG, Chen Y, Miao G, et al. The ER-localized transmembrane protein EPG-3/VMP1 regulates SERCA activity to control ER-isolation membrane contacts for autophagosome formation. Mol Cell 2017;67:974–989.e976.
37. Tabara LC, Escalante R. VMP1 establishes ER-microdomains that regulate membrane contact sites and autophagy. PLoS One 2016;11:e0166499.
38. Baselli GA, Jamialahmadi O, Pelusi S, et al. Rare ATG7 genetic variants predispose patients to severe fatty liver disease. J Hepatol 2022;77:596–606.
39. Ni HM, Woolbright BL, Williams J, et al. Nrf2 promotes the development of fibrosis and tumorigenesis in mice with defective hepatic autophagy. J Hepatol 2014;61:617–625.
40. Khambu B, Huda N, Chen X, et al. HMGB1 promotes ductular reaction and tumorigenesis in autophagy-deficient livers. J Clin Invest 2018;128:2419–2435.
41. Wang X, Wang H, Xu B, et al. Receptor-mediated ER export of lipoproteins controls lipid homeostasis in mice and humans. Cell Metab 2021;33:350–366.e357.
42. Wang Y, Liu L, Zhang H, et al. Mea6 controls VLDL transport through the coordinated regulation of COPII assembly. Cell Res 2016;26:787–804.
43. Tang VT, McCormick J, Xu B, et al. Hepatic inactivation of murine Surf4 results in marked reduction in plasma cholesterol. Elife 2022;11:e82269.
44. Danpanichkul P, Suparan K, Kim D, Wijarnpreecha K. What is new in metabolic dysfunction-associated steatotic liver disease in lean individuals: from bench to bedside. J Clin Med 2024;13:278.
45. Longo M, Meroni M, Paolini E, et al. TM6SF2/PNPLA3/MBOAT7 loss-of-function genetic variants impact on NAFLD development and progression both in patients and in in vitro models. Cell Mol Gastroenterol Hepatol 2022;13:759–788.
46. Chouik Y, Di Filippo M, Radenne S, et al. Combination of heterozygous APOB gene mutation with PNPLA3 and TM6SF2 variants promotes steatotic liver disease, cirrhosis and HCC development. Liver Int 2024;44:1474–1477.
47. Burnett JR, Zhong S, Jiang ZG, et al. Missense mutations in APOB within the betaalpha1 domain of human APOB-100 result in impaired secretion of ApoB and ApoB-containing lipoproteins in familial hypobetalipoproteinemia. J Biol Chem 2007;282:24270–24283.
48. Ma X, Wei X, Niu M, et al. Genetic disruption of mitochondrial dynamics and stasis leads to liver injury and tumorigenesis. J Clin Invest 2025;136:e194441.
49. Li Y, Chao X, Yang L, et al. Impaired fasting-induced adaptive lipid droplet biogenesis in liver-specific Atg5-deficient mouse liver is mediated by persistent nuclear factor-like 2 activation. Am J Pathol 2018;188:1833–1846.
50. Chao X, Wang S, Ma X, et al. Persistent mTORC1 activation due to loss of liver tuberous sclerosis complex 1 promotes liver injury in alcoholic hepatitis. Hepatology 2023;78:503–517.
51. Wang S, Ni HM, Chao X, et al. Critical role of TFEB-mediated lysosomal biogenesis in alcohol-induced pancreatitis in mice and humans. Cell Mol Gastroenterol Hepatol 2020;10:59–81.
52. Chao X, Wang S, Zhao K, Li Y, et al. Impaired TFEB-mediated lysosome biogenesis and autophagy promote chronic ethanol-induced liver injury and steatosis in mice. Gastroenterology 2018;155:865–879.e812.
53. Chao X, Wang S, Fulte S, Ma X, et al. Hepatocytic p62 suppresses ductular reaction and tumorigenesis in mouse livers with mTORC1 activation and defective autophagy. J Hepatol 2022;76:639–651.
54. Wieckowski MR, Giorgi C, Lebiedzinska M, et al. Isolation of mitochondria-associated membranes and mitochondria from animal tissues and cells. Nat Protoc 2009;4:1582–1590.

Received August 5, 2025. Accepted January 27, 2026.

Correspondence
Address correspondence to: Hong-Min Ni, MD, Department of Pharmacology, Toxicology and Therapeutics, University of Kansas Medical Center, MS 1018, 3901 Rainbow Boulevard, Kansas City, Kansas 66160. e-mail: hni@kumc.edu.

Acknowledgments
The authors acknowledge KUMC Liver Center for human liver samples. The authors also thank KUMC Electron Microscopy Research Lab facility for assistance with the transmission electron microscope.

CRediT Authorship Contributions

Allen Chen, PhD (Conceptualization: Supporting; Data curation: Lead; Formal analysis: Equal; Investigation: Equal; Methodology: Equal; Writing – original draft: Lead)

Khue Nguyen (Data curation: Equal; Formal analysis: Supporting; Writing – review & editing: Equal)

Mengwei Niu, PhD (Data curation: Equal; Formal analysis: Supporting; Writing – review & editing: Equal)

Xiaoxiao Jiang, PhD (Data curation: Supporting; Formal analysis: Supporting; Writing – review & editing: Supporting)

Xiaochen Yu, PhD (Data curation: Supporting; Formal analysis: Supporting; Writing – review & editing: Supporting)

Yan Xie (Data curation: Supporting; Formal analysis: Supporting; Writing – review & editing: Supporting)

Wanqing Liu, PhD (Conceptualization: Supporting; Funding acquisition: Supporting; Writing – original draft: Supporting; Writing – review & editing: Supporting)

Nicholas O. Davidson, MD (Conceptualization: Equal; Data curation: Supporting; Formal analysis: Supporting; Funding acquisition: Supporting; Writing – original draft: Supporting; Writing – review & editing: Equal)

Wen-Xing Ding, PhD (Conceptualization: Equal; Formal analysis: Equal; Funding acquisition: Supporting; Writing – original draft: Supporting; Writing – review & editing: Equal)

Hong-Min Ni, MD (Conceptualization: Lead; Data curation: Equal; Formal analysis: Equal; Funding acquisition: Lead; Investigation: Lead; Methodology: Equal; Supervision: Lead; Validation: Equal; Writing – original draft: Equal; Writing – review & editing: Lead)

Conflicts of interest
The authors disclose no conflicts.

Funding
The research was supported in part by National Institute of Diabetes and Digestive and Kidney Diseases R56DK129234 and R01DK134737 (to Hong-Min Ni); National Institute on Alcohol Abuse and Alcoholism R37AA020518 and R01AA031230 (to Wen-Xing Ding); DK119437, HL151328, and DDRCC P30 DK052574 (to Nicholas O. Davidson); National Institute of Diabetes and Digestive and Kidney Diseases R01 DK124612 and National Institute of Environmental Health Services R01 ES034410 (to Wanqing Liu); and Allen Chen was partially supported by T32 DK128770.

Cellular and Molecular Gastroenterology and Hepatology, Volume *20*

Supplemental information

Overlapping Yet Distinct Functions of Vacuole Membrane Protein 1 and Transmembrane Protein 41B in Modulating Hepatic Lipoprotein Secretion and Autophagy

Allen Chen, Khue Nguyen, Mengwei Niu, Xiaoxiao Jiang, Xiaochen Yu, Yan Xie, Wanqing Liu, Nicholas O. Davidson, Wen-Xing Ding, and Hong-Min Ni

Figure 1F

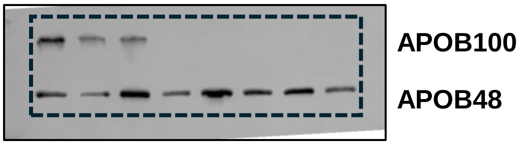

Figure 2E

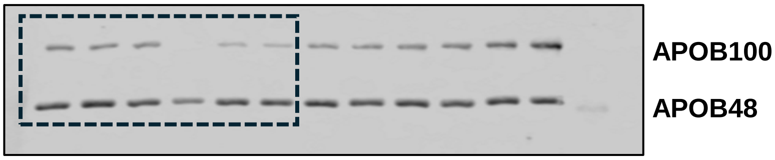

Figure 3F

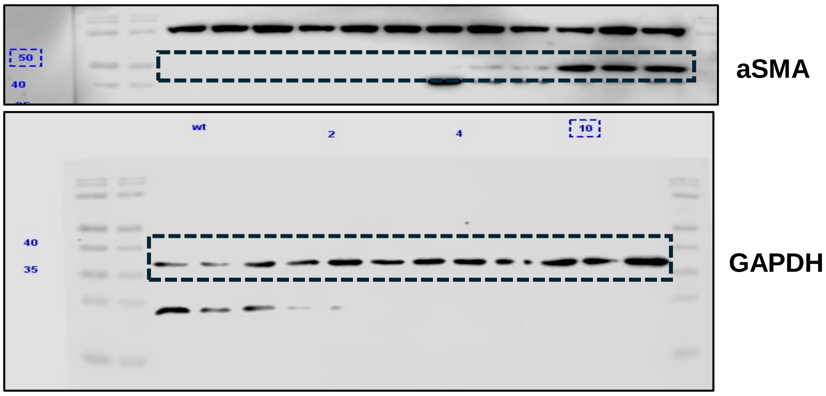

Figure 4A

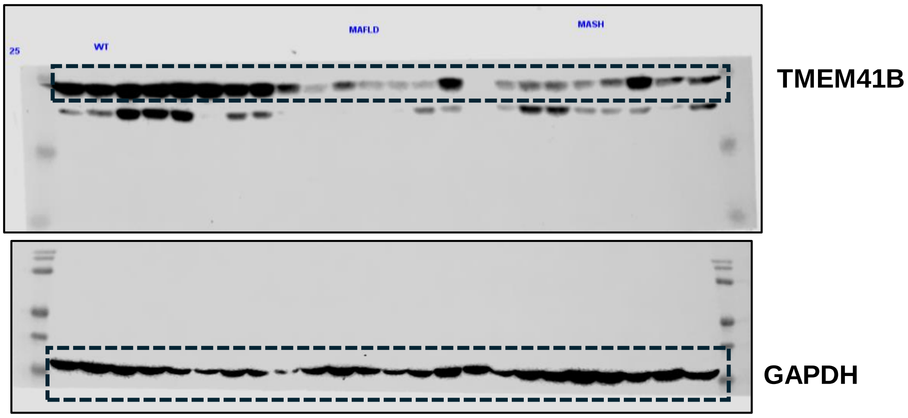

Figure 4C

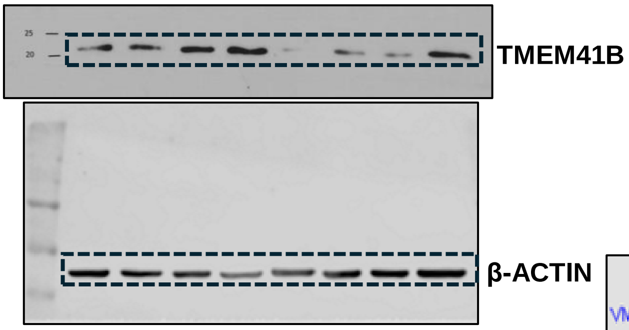

Figure 4G

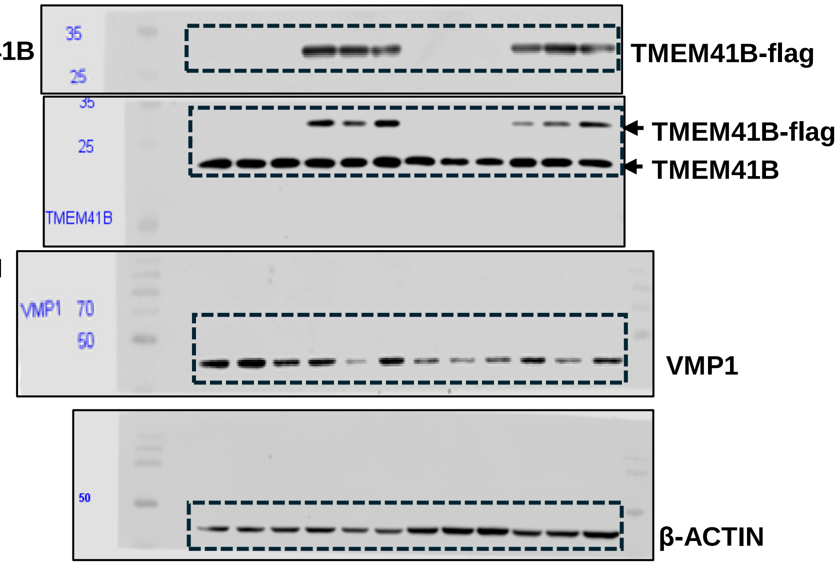

Figure 7D

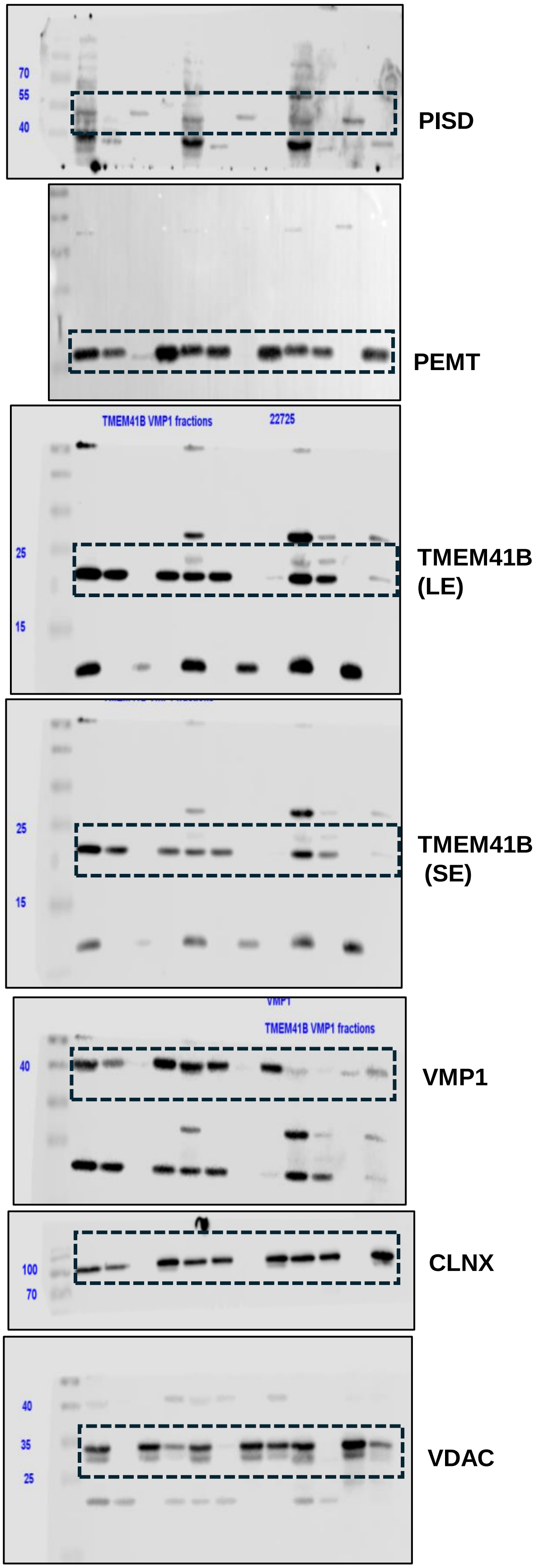

Figure 8E

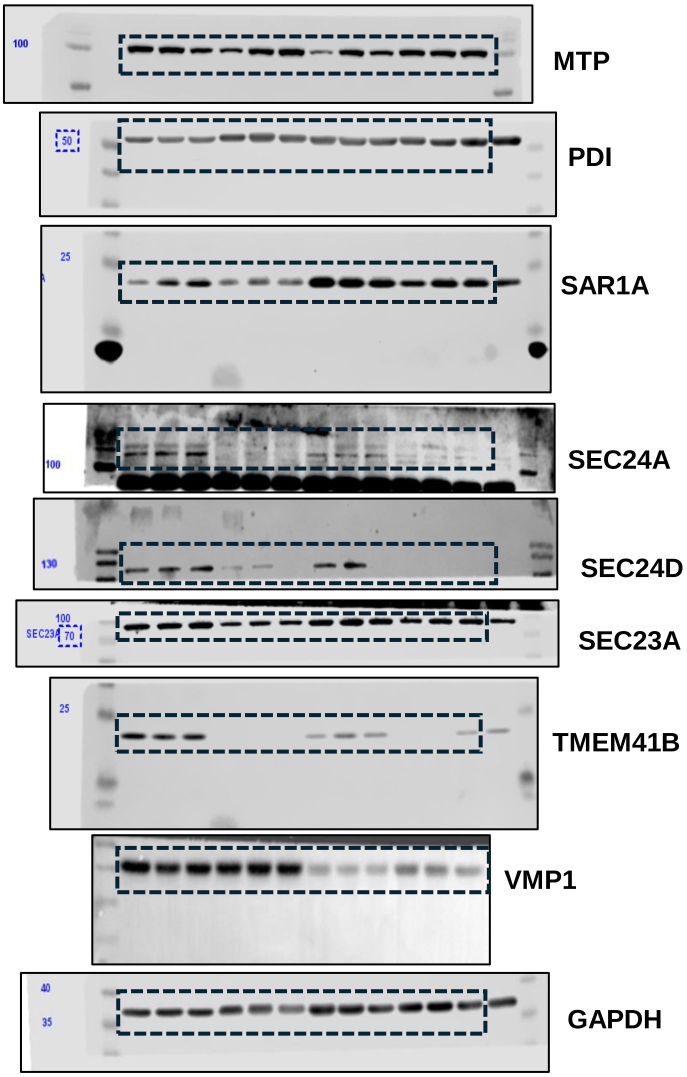

Figure 8F

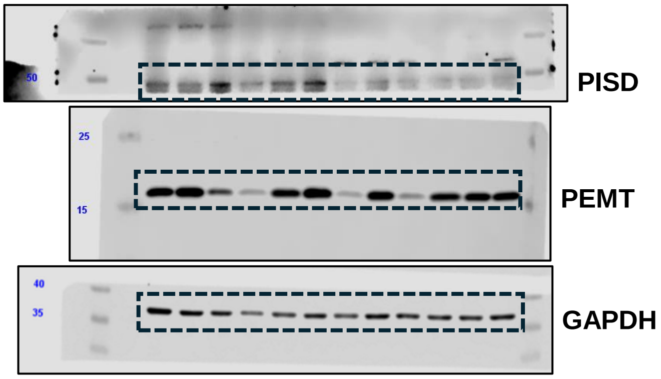

Figure 12A

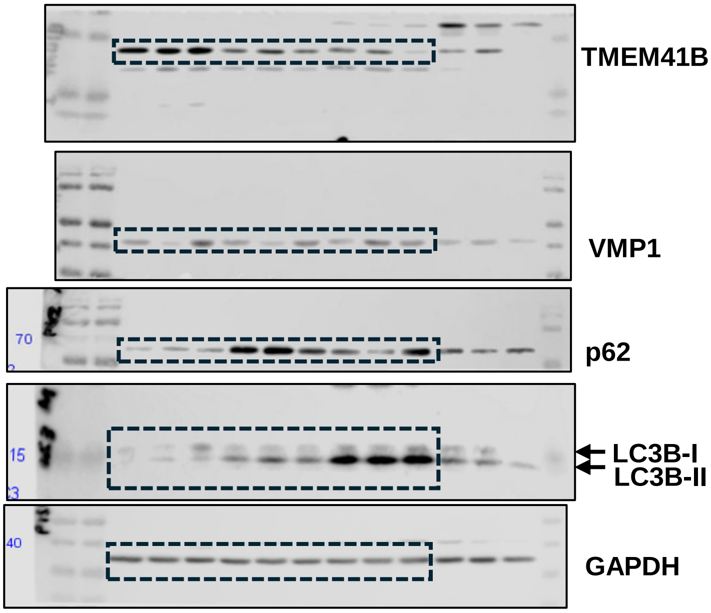

Figure 12B

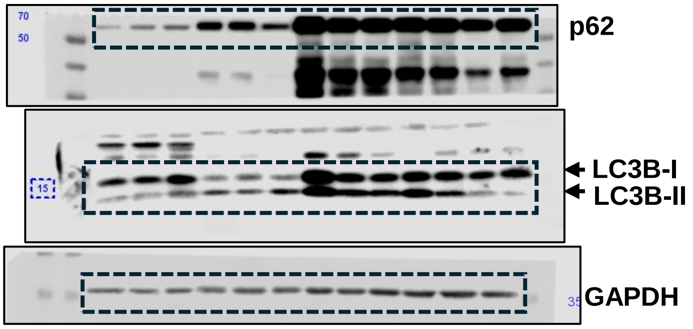

Figure 12D

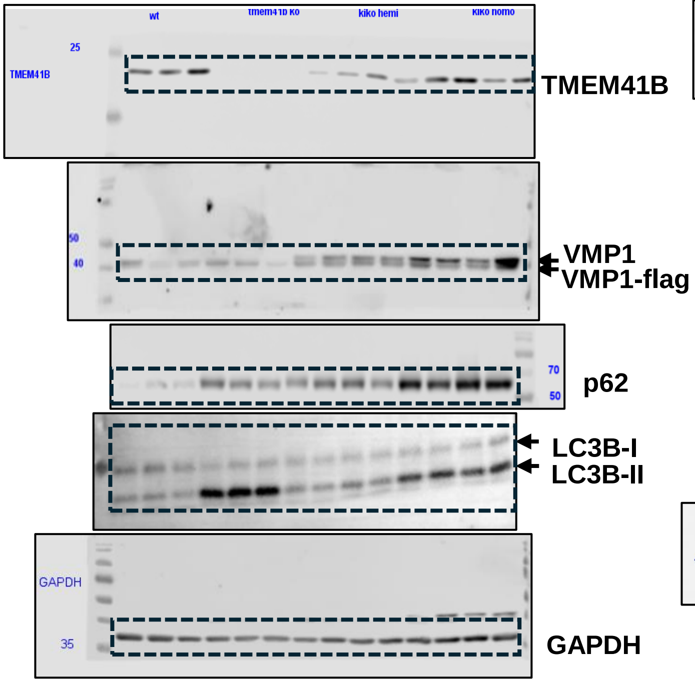

Figure 12E

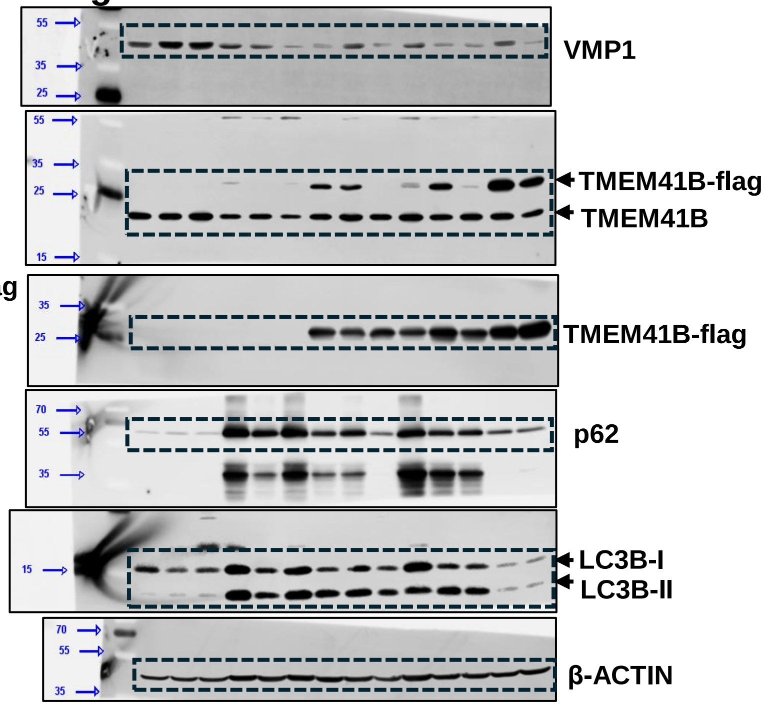

Supplement: Extended PDF [file mmc2.pdf]
